# Assessment of ferroptosis-associated gene signatures as potential biomarkers for differentiating latent from active tuberculosis in children

**DOI:** 10.1099/mgen.0.000997

**Published:** 2023-05-10

**Authors:** Liang Chen, Jie Hua, Xiaoting Dai, Xiaopu He

**Affiliations:** ^1^​ Department of Infectious Diseases, Nanjing Lishui People’s Hospital, Zhongda Hospital Lishui Branch, Southeast University, Nanjing, PR China; ^2^​ Department of Gastroenterology, Liyang People’s Hospital, Liyang Branch Hospital of Jiangsu Province Hospital, Nanjing, PR China; ^3^​ Department of Geriatric Gastroenterology, The First Affiliated Hospital of Nanjing Medical University, Nanjing, PR China

**Keywords:** ferroptosis, gene, children, active tuberculosis, latent tuberculosis infection, biomarkers

## Abstract

Ferroptotic cell death is a regulated process that is governed by iron-dependent membrane lipid peroxide accumulation that plays a pathogenic role in several disease-related settings. The use of ferroptosis-related genes (FRGs) to distinguish active tuberculosis (ATB) from latent tuberculosis infection (LTBI) among children, however, remains to be analysed. Tuberculosis-related gene expression data and FRG lists were obtained, respectively, from Gene Expression Omnibus (GEO) and FerrDb. Differentially expressed FRGs (DE-FRGs) detected when comparing samples from paediatric ATB and LTBI patients were explored using appropriate bioinformatics techniques, after which enrichment analyses were performed for these genes and hub genes were identified, with these genes then being used to explore potential drug interactions and construct competing endogenous RNA (ceRNA) networks. The GSE39939 dataset yielded 124 DE-FRGs that were primarily related to responses to oxidative, chemical and extracellular stimulus-associated stress. In total, the LASSO and SVM-RFE algorithms enabled the identification of nine hub genes (*MAPK14*, *EGLN2*, *IDO1*, *USP11*, *SCD*, *CBS*, *PARP8*, *PARP16*, *CDC25A*) that exhibited good diagnostic utility. Functional enrichment analyses of these genes suggested that they may govern ATB transition from LTBI through the control of many pathways, including the immune response, DNA repair, transcription, RNA degradation, and glycan and energy metabolism pathways. The CIBERSORT algorithm suggested that these genes were positively correlated with inflammatory and myeloid cell activity while being negatively correlated with the activity of lymphocytes. A total of 50 candidate drugs targeting 6 hub DE-FRGs were also identified, and a ceRNA network was used to explore the complex interplay among these hub genes. The nine hub FRGs defined in this study may serve as valuable biomarkers differentiating between ATB and LTBI in young patients.

## Data Summary

Two publicly available datasets were analysed in this study. These data can be found in the GSE39939 (https://www.ncbi.nlm.nih.gov/geo/query/acc.cgi?acc=GSE39939) and GSE39940 (https://www.ncbi.nlm.nih.gov/geo/query/acc.cgi?acc=GSE39940) datasets.

Figshare data – https://doi.org/10.6084/m9.figshare.22105139.v1 [1].

**Impact Statement**

Establishing reliable biomarkers that can distinguish active tuberculosis (ATB) from latent tuberculosis infection (LTBI) in children is urgent in terms of both appropriate treatment and the control of Mtb spread. Ferroptosis is a newly discovered process that has a potential role in the control of Mtb infection. However, most studies have been performed in animal models and data on ferroptosis in relation to Mtb infection in clinical samples are scarce, particularly in paediatrics. In this study, we used multiple bioinformatics approaches and identified nine ferroptosis-related gene signatures as biomarkers for accurate differentiation between ATB and LTBI in children. Our study also offers insights into the molecular mechanisms by which childhood LTBI progresses to ATB.

## Introduction

Tuberculosis (TB), which is an infection mediated by *Mycobacterium tuberculosis* (Mtb), is the deadliest pathogen-related cause of death in the world and one of the leading global causes of human mortality [2]. An estimated 500 000–1 000 000 children are affected by TB each year, of whom 226 000 die [3]. Of an estimated 2–3 billion individuals infected by Mtb, 5–15 % are expected to develop TB at some point in their lives, with this risk being most pronounced among young children [4, 5]. The treatment of TB can be a complex and prolonged process, resulting in poor patient compliance, particularly in paediatric populations. The factors that ultimately govern the transition between active TB (ATB) and latent TB infection (LTBI) remain to be fully clarified, and the clinical differentiation between these two disease states remains challenging, despite being critical to providing patients with appropriate treatments aimed at curtailing further TB spread. The two approaches most frequently used to assess TB infection are the interferon-γ release assay (IGRA) and the tuberculin skin test (TST), but neither can distinguish between ATB and LTBI [6]. In children with TB suffering from malnourishment or human immunodeficiency virus (HIV) infection, TST or IGRA results may be non-reactive, while TSTs often shows false positives in BCG-vaccinated individuals [7], especially atypical manifestations in many cases, which complicate and delay TB diagnosis. There is thus a pressing need for the identification of alternative biomarkers that can reliably detect the different forms of TB infection in children.

Death of the host cells plays a critical role in controlling the progression of LTBI to ATB [8–10]. Several gene signatures have been identified that not only reveal the pathogenic mechanism but can also be used as novel biomarkers for distinguishing ATB from LTBI [8–11]. Ferroptotic cell death is a recently discovered process that is driven by the accumulation of excessively high iron levels within cells, contributing to lipid peroxidation and the lethal rupture of the cell membrane [12]. A potential role for ferroptosis in the Mtb-mediated induction of cell death has recently been described [13]. Specifically, the replication of Mtb within host macrophage cells can contribute to intracellular labile iron accumulation, together with higher levels of mitochondrial superoxide generation, lipid peroxidation and necrotic cell death. Notably, Mtb-infected macrophages exhibited increases in glutathione peroxidase 4 (GPX4) expression and glutathione (GSH) levels, which are closely associated with ferroptotic death. When these cells are treated with iron chelators or the lipid peroxidation inhibitor ferrostatin-1 (Fer-1), this can protect against lung tissue death while lowering the overall mycobacterial burden [13].

However, as most of the studies have been performed in animal models, data on ferroptosis in Mtb-infected clinical samples are scarce, particularly in paediatrics. In this study, the effectiveness of ferroptosis-related gene (FRG) signatures as biomarkers for the differentiation of ATB and LTBI in children was analysed, and a bioinformatics approach was used to explore the relationship between these genes and immune cell populations.

## Methods

### Data source

For the present study, data were downloaded from the National Center for Biotechnology Information (NCBI) GEO database (http://www.ncbi.nlm.nih.gov/geo). Eligible data were derived from studies of children under 15 years of age who were HIV-negative, with all sample collection having been performed before anti-mycobacterial treatment was initiated. These criteria were used to select the two largest datasets for analysis. The GSE39939 microarray training dataset comprised whole-blood samples from 52 and 14 Kenyan paediatric ATB and LTBI patients, respectively. The GSE39940 microarray validation dataset comprised whole-blood samples from 52 and 54 paediatric South African and Malawian ATB and LTBI patients, respectively. The latter of these two data datasets was used for hub gene validation. All patients with ATB had been diagnosed based on the confirmed isolation and culture of Mtb derived from respiratory samples together with clinical symptoms consistent with TB, or negative Mtb cultures together with clinical and radiological findings consistent with ATB. LTBI was diagnosed based on confirmed contact with individuals with positive TB smear results together with positive TST or IGRA results in the absence of any clinical or radiological signs of ATB on follow-up.

In total, 728 FRGs identified using FerrDb were analysed (File S1, available in the online version of this article). Potential drug interactions were explored with the Drug Gene Interaction Database (DGIdb), with the DrugBank database serving as a source of structural information regarding drugs predicted to target hub DE-FRGs.

### Differential expression analyses

The relative expression of individual FRG was compared between the ATB and LTBI groups in the GSE39939 dataset. DE-FRGs were identified using the ‘limma’ package in R, based on *P-*values <0.05 when compared via Student’s *t*-test analyses after determining the normality of the data distribution (File S2).

### Functional enrichment analyses

Gene Ontology (GO) and Kyoto Encyclopedia of Genes and Genomes (KEGG) enrichment analyses were performed with the R ‘clusterprofiler’ package, with an FDR <0.05 and an adjusted *P-*value <0.05 as the significance threshold. GO terms were classified into the biological process (BP), molecular function (MF) and cellular component (CC) categories. The top 10 enriched terms are shown in this study.

### Optimal diagnostic hub gene selection

The glmnet package was utilized to implement a LASSO algorithm to reduce data dimensionality, with DE-FRGs identified when comparing LTBI and ATB samples being retained for feature selection, and biomarkers of ATB then being selected through this algorithmic approach. The SVM package was additionally utilized to implement a support vector machine recursive feature elimination (SVM-RFE) model with 10-fold cross-validation. Optimal ATB-related marker genes were selected based on the overlap in gene sets generated by these two algorithmic strategies, and the diagnostic utility of these individual hub DE-FRGs was examined based on the use of the area under the receiver operating characteristic (ROC) curve (AUC) with corresponding analyses of accuracy, sensitivity and specificity from the GSE39939 dataset. A logistic regression model was further constructed using the combination of nine hub DE-FRGs to predict sample classification in the GSE39940 dataset using the ‘glm’ package in R, and ROC curves were used to validate the diagnostic utility of the model.

### Immune cell analyses

The CIBERSORT algorithm [14] was leveraged to examine the expression of 22 different immune cell populations in samples from the GSE39939 dataset, and violin plots were used to represent the resultant data (Supplementary file 3). Associations between hub FRGs and immune cell populations were assessed with Spearman correlation analyses, and results were visualized with the ‘corrplot’ package.

### Single-gene-set enrichment analysis

The R GSEA (v.4.1.0) package was employed for single-sample gene set enrichment analysis (ssGSEA) analyses exploring pathways associated with hub FRGs by examining correlations between these genes and all other genes included within the GSE39939 dataset. These genes were then rank ordered based on the strength of these correlative relationships and served as the testing gene set, while the KEGG signalling pathway gene set was selected as a target for enrichment analyses within this gene set.

### Single-gene-set variation enrichment analysis

The R GSVA package was used for gene set variation analysis (GSVA) analyses of each hub gene, using the KEGG pathway gene set as a background. Using the limma package, differences in GSVA scores for marker genes in the low- and high-expression groups were compared. Differences were screened with |*t*|>2 and *P*<0.05 as significance criteria, with *t*>0 and *t*<0 being indicative of pathway activation in the high and low-expression groups, respectively.

### ceRNA network development

Interacting miRNAs associated with the identified hub DE-FRGs were detected with starBase. The mRNA sequences for these hub DE-FRGs were also downloaded from the NCBI, and miRbase was used to download human miRNA sequences, after which the TargetScan, miRDB and miRanda databases were used to predict miRNA target genes (File S4). StarBase was also used to screen for mRNA–lncRNA interactions (File S5), allowing for the construction of an mRNA–miRNA–lncRNA network.

### Statistical analyses

Data were compared between groups with Student’s *t*-tests, while Pearson correlation analyses were employed when assessing associations among DE-FRGs. The Jvenn package was used for Venn diagram construction. All ceRNA networks were visualized with Cytoscape. R (v 4.2.0) was employed for statistical analyses, and *P*<0.05 was the significance threshold.

## Results

### DE-FRG identification

**Fig. 1.**
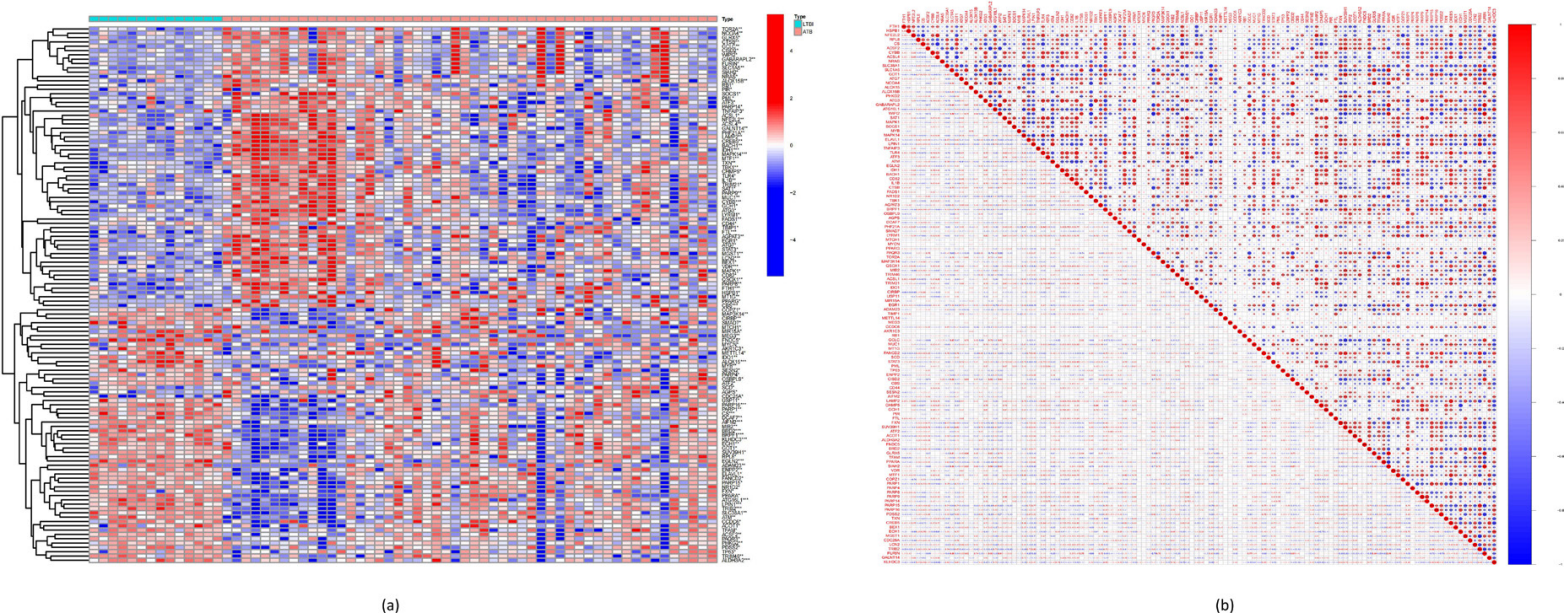
Expression levels of DE-FRGs in ATB and LTBI. (**a**) Heatmap showing the expression patterns of the DE-FRGs across samples. (**b**) Pearson correlation coefficients of these genes.

In total, 124 FRGs were found to be differentially expressed when comparing LTBI and ATB samples in the GSE39939 dataset, of which 67 and 57 were upregulated and downregulated in ATB, respectively (Fig. 1a and File S2). Correlations among these genes are shown in Fig. 1b.

### Functional DE-FRG analyses

**Fig. 2.**
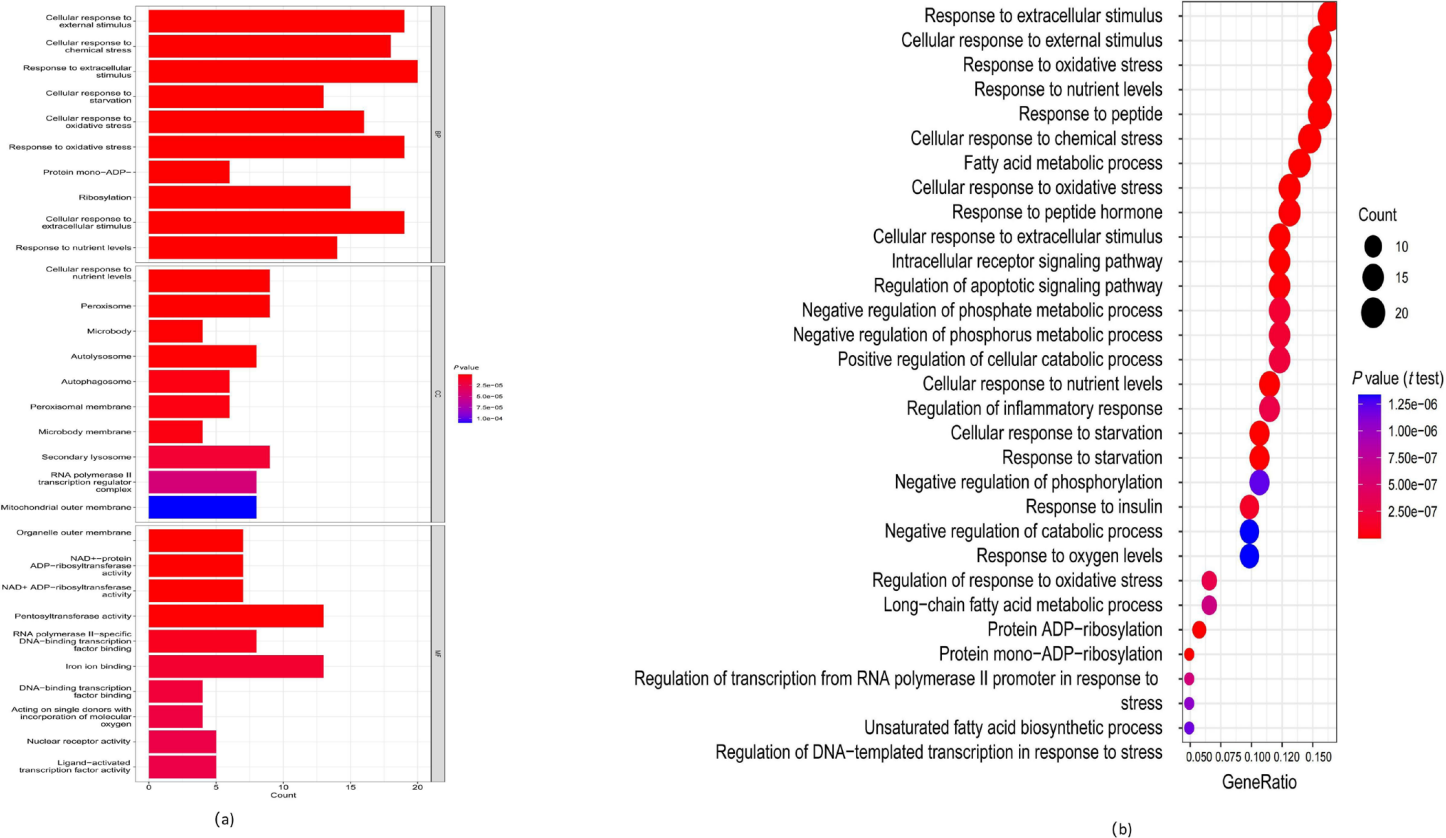
Functional analyses of the DE-FRGs. (**a**) GO enrichment and (**b**) KEGG analyses indicated that the DE-FRGs were significantly associated with pathways involving oxidative stress, chemical stress, nutrient levels, extracellular stimuli and the regulation of autophagy (Fisher’s test).

GO and KEGG enrichment analyses were performed to better understand the biological roles played by the DE-FRGs in the context of paediatric ATB compared with LTBI. The DE-FRGs were found to be primarily associated with GO terms related to responses to oxidative stress, chemical stress, extracellular stimuli and nutrient levels (Fig. 2a), and with similar KEGG pathways (Fig. 2b). The findings thus suggested that ATB in children is associated with oxidative and chemical stress responses, nutrient levels and extracellular stimuli, in comparison with LTBI.

### Identification of key DE-FRGs associated with paediatric ATB and LTBI

**Fig. 3.**
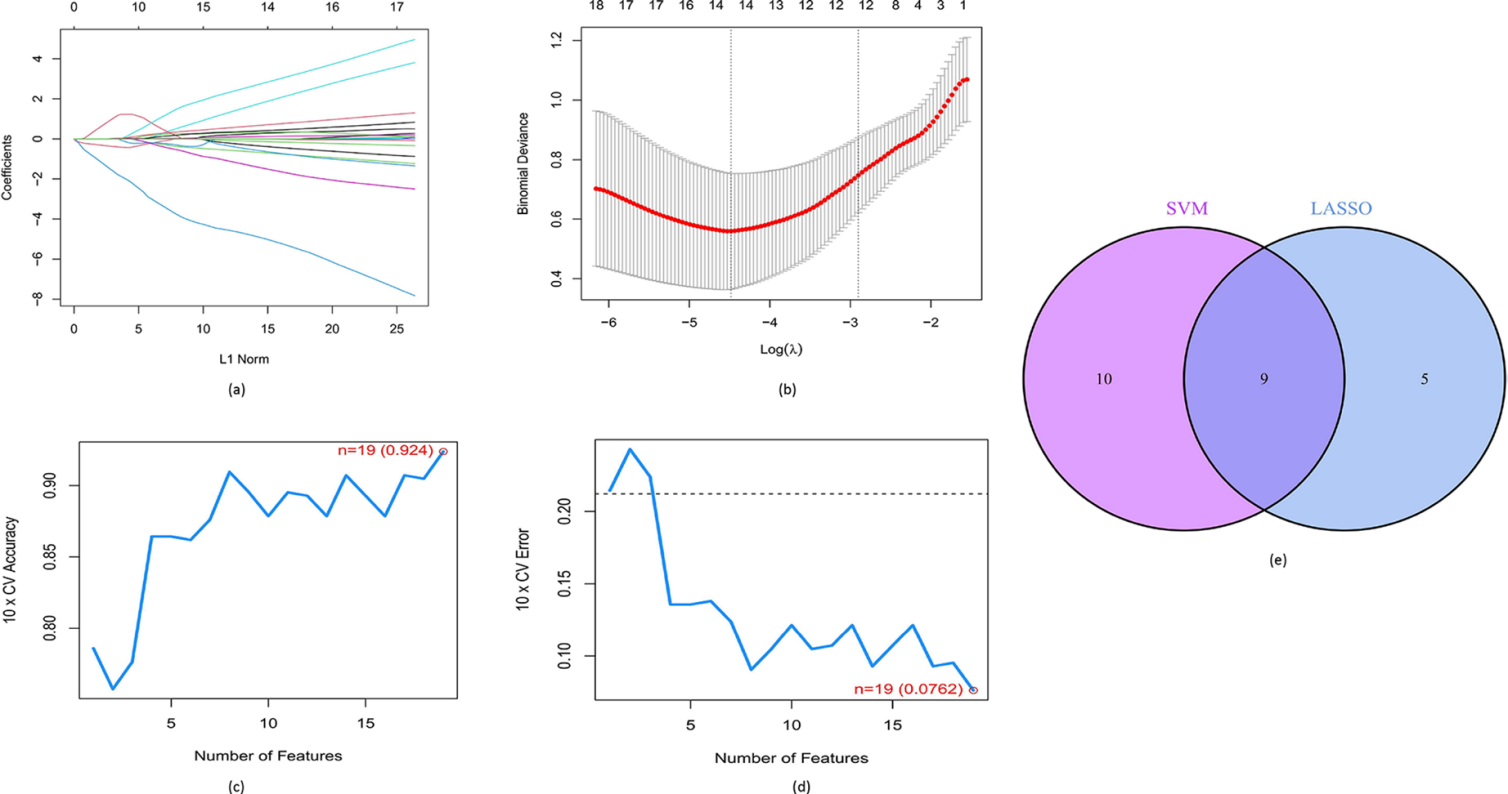
Nine hub DE-FRGs were identified as diagnostic genes for the progression of ATB from LTBI. (**a, b**) LASSO logistic regression, with penalty parameter tuning conducted by 10-fold cross-validation was used to select 14 ATB-related features. (**c, d**) The SVM-RFE algorithm was used to filter the 19 DE-FRGs to identify the optimal combinations of specific genes. Finally, nine genes (maximal accuracy, 0.924; minimal RMSE, 0.0762) were identified as the optimal genes. (**e**) Marker genes obtained from the LASSO and SVM-RFE models.

Using the GSE39939 training dataset, the LASSO and SVM-RFE machine learning algorithms were next implemented to identify hub DE-FRGs capable of reliably differentiating between paediatric LTBI and ATB patients. In LASSO analyses with 10-fold cross-validation-based penalty parameter tuning, 14 DE-FRGs were selected (Fig. 3a, b), while the SVM-RFE algorithm identified 19 optimal genes [maximal accuracy =0.924, minimal root mean square error (RMSE) = 0.0762] (Fig. 3c, d). A comparison of these two gene sets yielded a list of nine candidate hub DE-FRGs (*MAPK14*, *EGLN2*, *IDO1*, *USP11*, *SCD*, *CBS*, *PARP8*, *PARP16* and *CDC25A*) that were retained for downstream analyses (Fig. 3e).

**Fig. 4.**
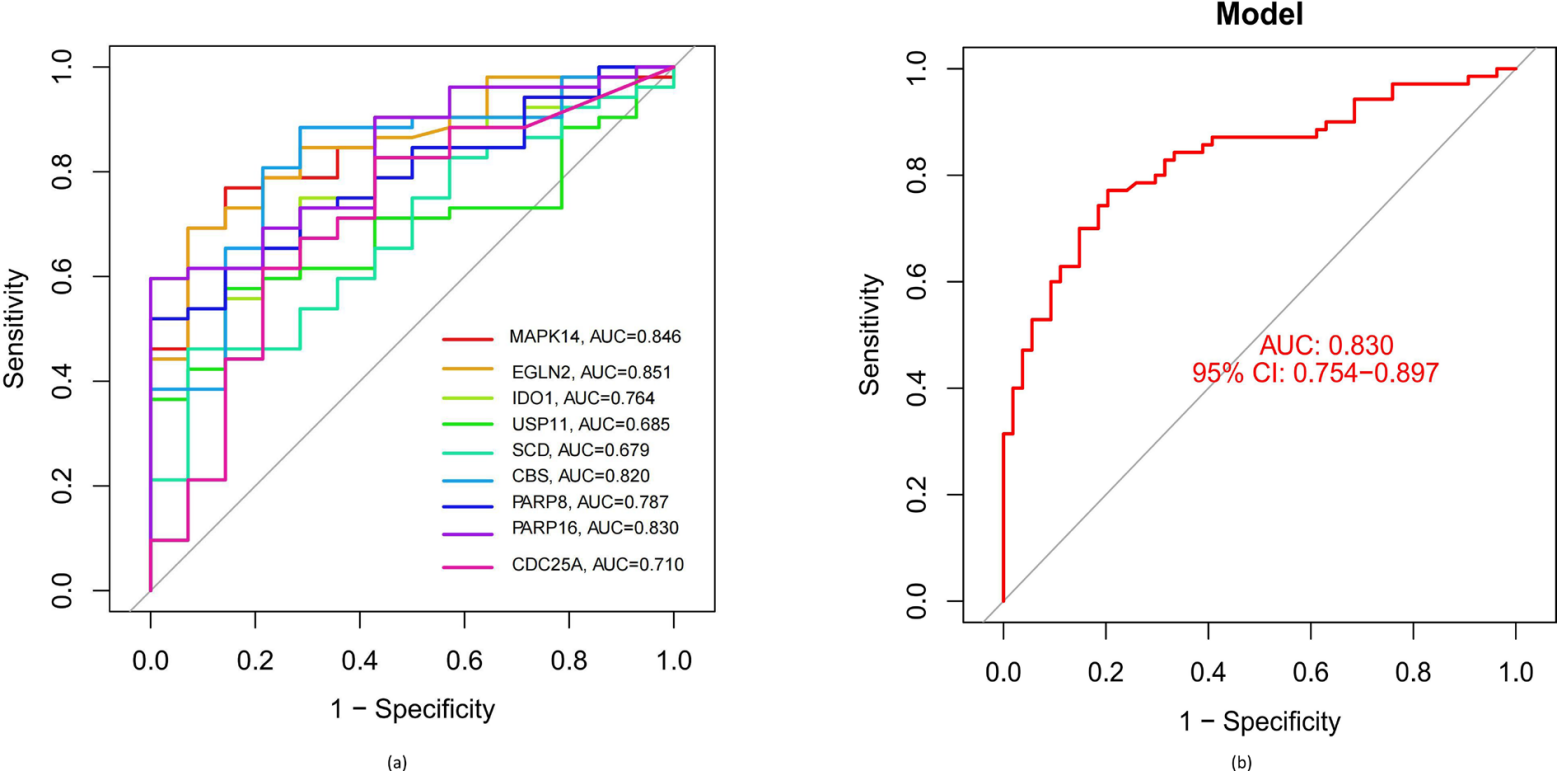
The use of nine hub DE-FRGs as tools for the differentiation between individuals with ATB and LTBI. (**a**) ROC curves for the nine individual marker genes from the GSE39939 dataset. (**b**) Logistic regression model for determining the AUC of differentiation between disease samples from the GSE39940 dataset using the combination of nine hub DE-FRGs.

**Fig. 5.**
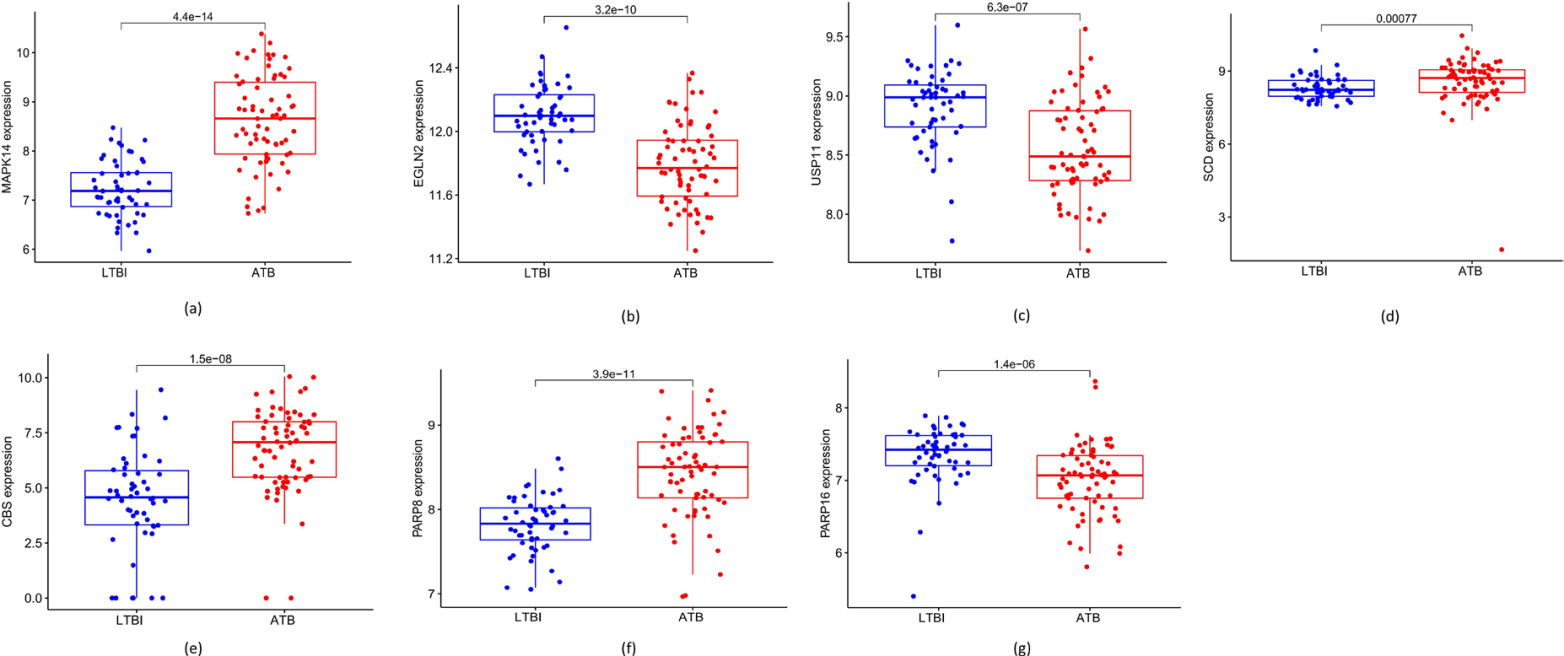
Expression of the marker genes in the validation dataset (GSE39940). The expression of *MAPK14* (**a**)*, CBS* (**e**), *SCD* (**d**) and *PARP8* (**f**) was increased, while that of *EGLN2* (**b**), *USP11* (**c**) and *PARP16* (**g**) was reduced in the ATB group compared with the LTBI group, consistent with the data from the GSE39930 training dataset.

ROC curves were next used to gain insight into how reliably the hub DE-FRGs could differentiate between LTBI and ATB samples from the GSE39939 training dataset, yielding individual AUC values ranging from 0.6 to 0.9 (Fig. 4a). After development of a logistic regression model using the R ‘glm’ package, it was found that the AUC value for the combination of the nine hub DE-FRGs was 0.830 in the GSE39940 test dataset (Fig. 4b), thus demonstrating that the model is both accurate and specific for individual genes when distinguishing between ATB and LTBI samples. In addition, the expression of the identified hub DE-FRGs was analysed in the GSE39940 dataset. This showed that the expression of *CBS* (*P*=1.5e−08), *MAPK14* (*P*=4.4e−14), *PARP8* (*P*=3.9e−11) and *SCD* (*P*=0.00077) was increased in samples from the ATB group, whereas the levels of *EGLN2* (*P*=3.2e–10), *PARP16* (*P*=1.4e–06) and *USP11* (*P*=6.3e–07) were reduced compared with LTBI samples, consistent with the data from the GSE39930 training dataset (Fig. 5).

### GSEA and GSVA analyses of identified hub DE-FRGs

**Fig. 6.**
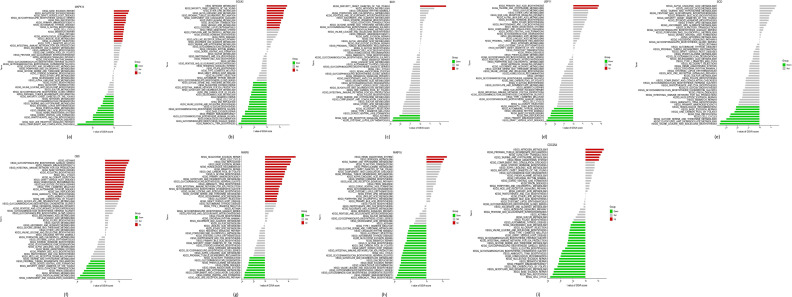
High- and low-expression groups based on the expression levels of each marker gene combined with GSVA. *PARP16* (**a**), *PARP8* (**b**), *CBS* (**c**), *SCD* (**d**), *USP11* (**e**), *IDO1*(f), *EGLN2* (**g**), *MAPK14* (**h**) and *CDC25A*(i).

To better understand the ability of the identified hub DE-FRGs to differentiate between samples from LTBI and ATB patients, a single-gene GSEA–KEGG pathway analysis was conducted. Strikingly, the majority of these genes were enriched in processes related to the immune response, DNA repair, transcription, RNA degradation, and carbohydrate and energy metabolism. GSVA analyses yielded similar results, indicating that high expression of *MAPK14*, *EGLN2* and *PARP16* may play a role in Mtb infection in children through the regulation of DNA repair and recombination, RNA degradation and the immune response (Table 1 and File S6). Similarly, GSVA analysis suggested that altered expression of the hub DE-FRGs induced ATB by regulating amino acid and glycan metabolism, DNA replication and the immune response (Fig. 6).

**Table 1.** Single-gene GSEA–KEGG pathway analysis for the nine hub DE-FRGs

Gene	Expression level in ATB	Enrichment pathways (top 3)
*PARP16*	Downregulation	KEGG_LYSINE_DEGRADATION KEGG_RNA_DEGRADATION KEGG_HOMOLOGOUS_RECOMBINATION
*PARP8*	Upregulation	KEGG_RNA_DEGRADATION KEGG_HOMOLOGOUS_RECOMBINATION KEGG_PROXIMAL_TUBULE_BICARBONATE_RECLAMATION
*CBS*	Upregulation	KEGG_PYRIMIDINE_METABOLISM KEGG_RNA_DEGRADATION KEGG_B_CELL_RECEPTOR_SIGNALING_PATHWAY
*SCD*	Upregulation	KEGG_HUNTINGTONS_DISEASE KEGG_OXIDATIVE_PHOSPHORYLATION KEGG_CARDIAC_MUSCLE_CONTRACTION
*USP11*	Downregulation	KEGG_RNA_DEGRADATION KEGG_HOMOLOGOUS_RECOMBINATION KEGG_N_GLYCAN_BIOSYNTHESIS
*IDO1*	Downregulation	KEGG_PYRIMIDINE_METABOLISM KEGG_SPLICEOSOME KEGG_INSULIN_SIGNALING_PATHWAY
*EGLN2*	Downregulation	KEGG_ADHERENS_JUNCTION KEGG_NON_HOMOLOGOUS_END_JOINING KEGG_RNA_DEGRADATION
*MAPK14*	Upregulation	KEGG_HUNTINGTONS_DISEASE KEGG_OXIDATIVE_PHOSPHORYLATION KEGG_RNA_DEGRADATION
*CDC25A*	Upregulation	KEGG_ADHERENS_JUNCTION KEGG_GLYCOSAMINOGLYCAN_BIOSYNTHESIS_KERATAN_SULFATE KEGG_TGF_BETA_SIGNALING_PATHWAY

### Relationship between immune cell populations and hub DE-FRGs

**Fig. 7.**
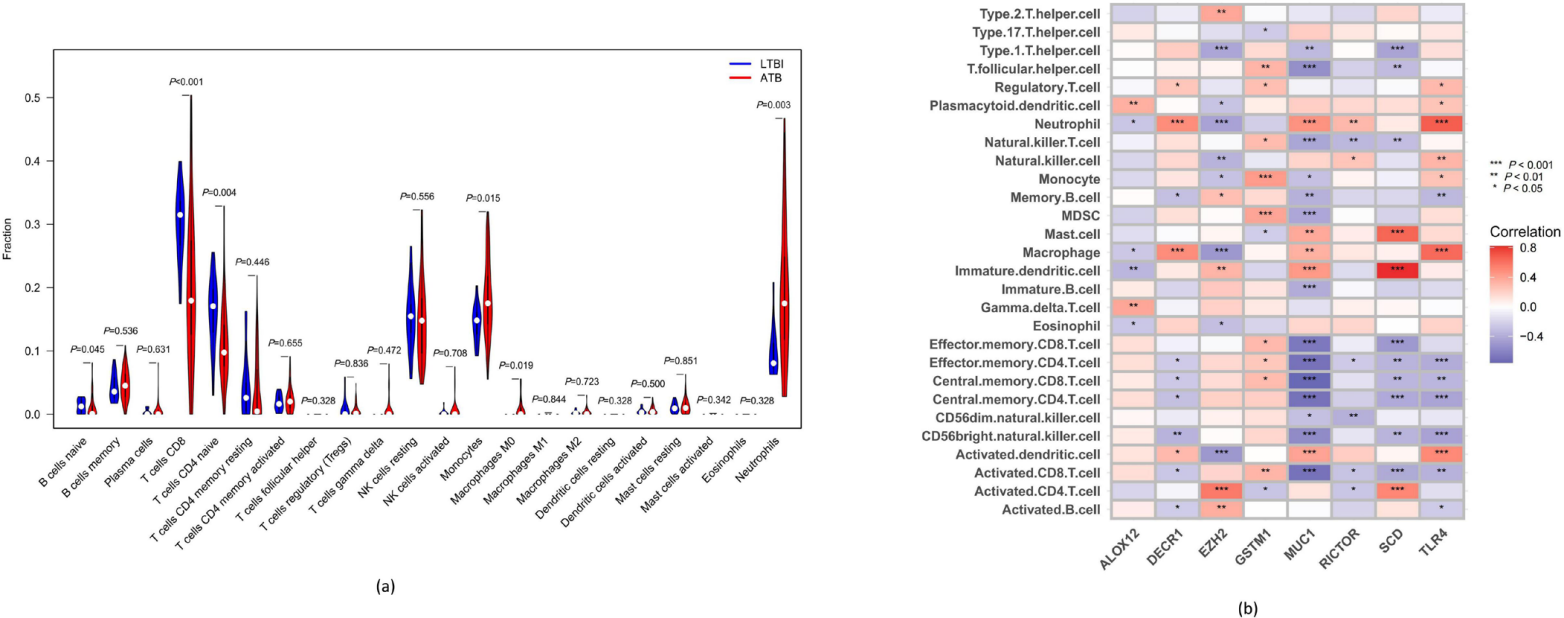
Immune landscape analysis. (**a**) The CIBERSORT algorithm was used to explore differences in the immune microenvironments between ATB and LTBI paediatric patients. (**b**) Pearson correlation analysis revealed that the expression levels of the hub DE-FRGss were positively correlated with myeloid and inflammatory cell populations but negatively correlated with lymphocyte levels.

The CIBERSORT algorithm [14] was next leveraged to compare predicted differences in immune cell populations between the whole-blood samples from paediatric LTBI and ATB patients. This analysis revealed that ATB patient samples contained larger inflammatory cell populations, including neutrophils, macrophages, monocytes and dendritic cells (DCs), while the opposite was true for lymphocyte populations, including naïve B cells, naïve CD4^+^ T cells and activated CD8^+^ T cells (Fig. 7a). Hub DE-FRG expression was negatively correlated with lymphocyte activity yet positively correlated with the activity of inflammatory and myeloid cells. For example, a positive correlation was detected between *MAPK14* expression and monocytes, neutrophils and M0 macrophages, whereas this gene was negatively correlated with CD8^+^ T cells. *PARP16* downregulation was negatively associated with naïve B cell and CD8^+^ T cell populations and positively correlated with monocyte and neutrophil populations. Moreover, *CBS* levels were positively correlated with monocytes, neutrophils and M0 macrophages, yet negatively related to naïve B cells and CD8^+^ T cells (Fig. 7b).

### Identification of candidate hub DE-FRG-targeting drugs

**Fig. 8.**
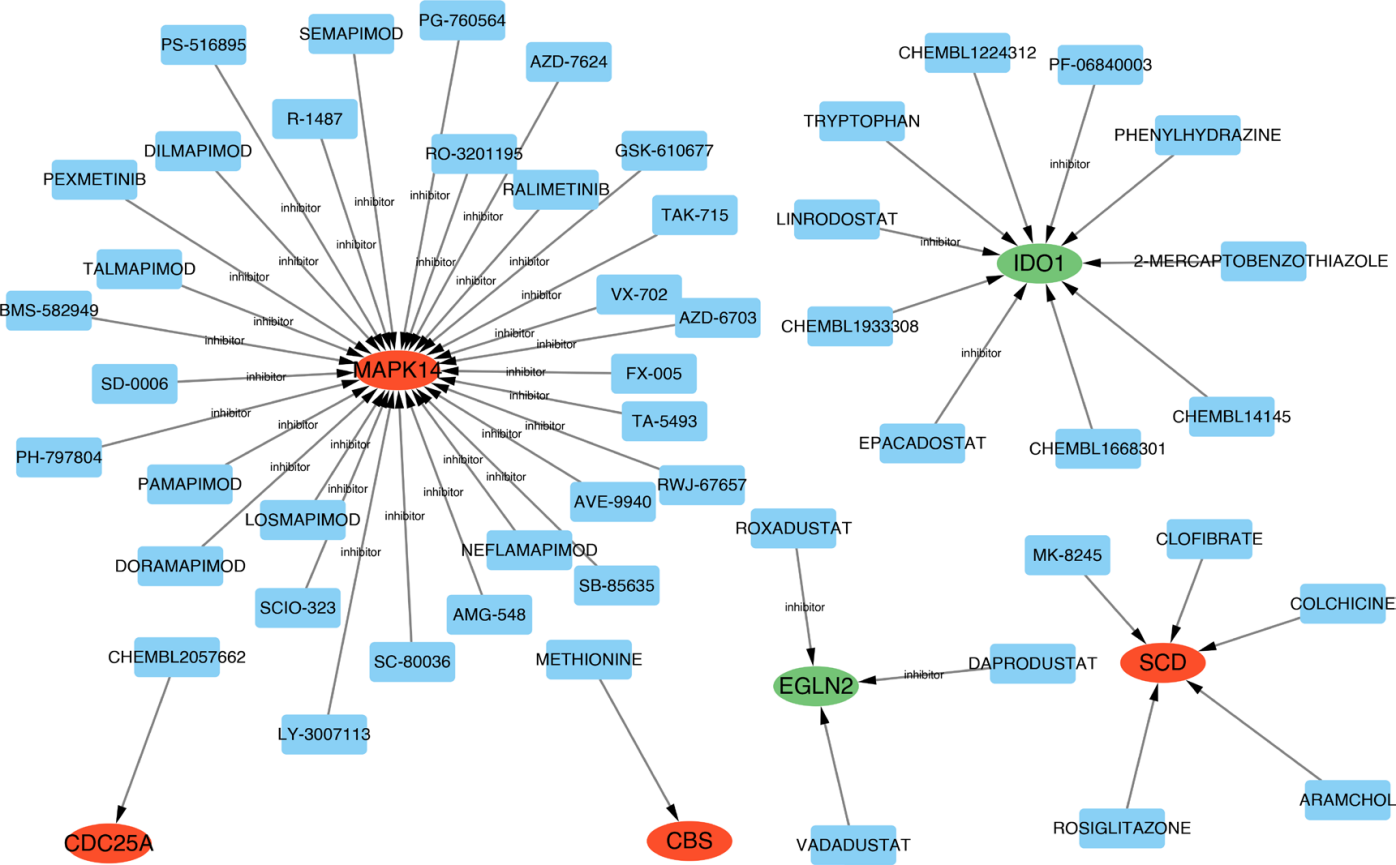
Prediction of marker gene-targeted drugs. Drugs that may target marker genes were identified using the DGIdb database, together with the associations between them. Red and green represent the expression levels of upregulated and downregulated genes, respectively, in ATB compared with LTBI.

Using DGIdb, 50 candidate drugs were identified and predicted to target 6 of the included hub DE-FRGs (*MAPK14*, *CDC25A*, *CBS*, *SCD*, *EGLN2*, *IDO1*) when using default interaction parameters. Cytoscape was used to visualize the resultant data (Fig. 8).

### Establishment of a hub DE-FRG-based ceRNA network

**Fig. 9.**
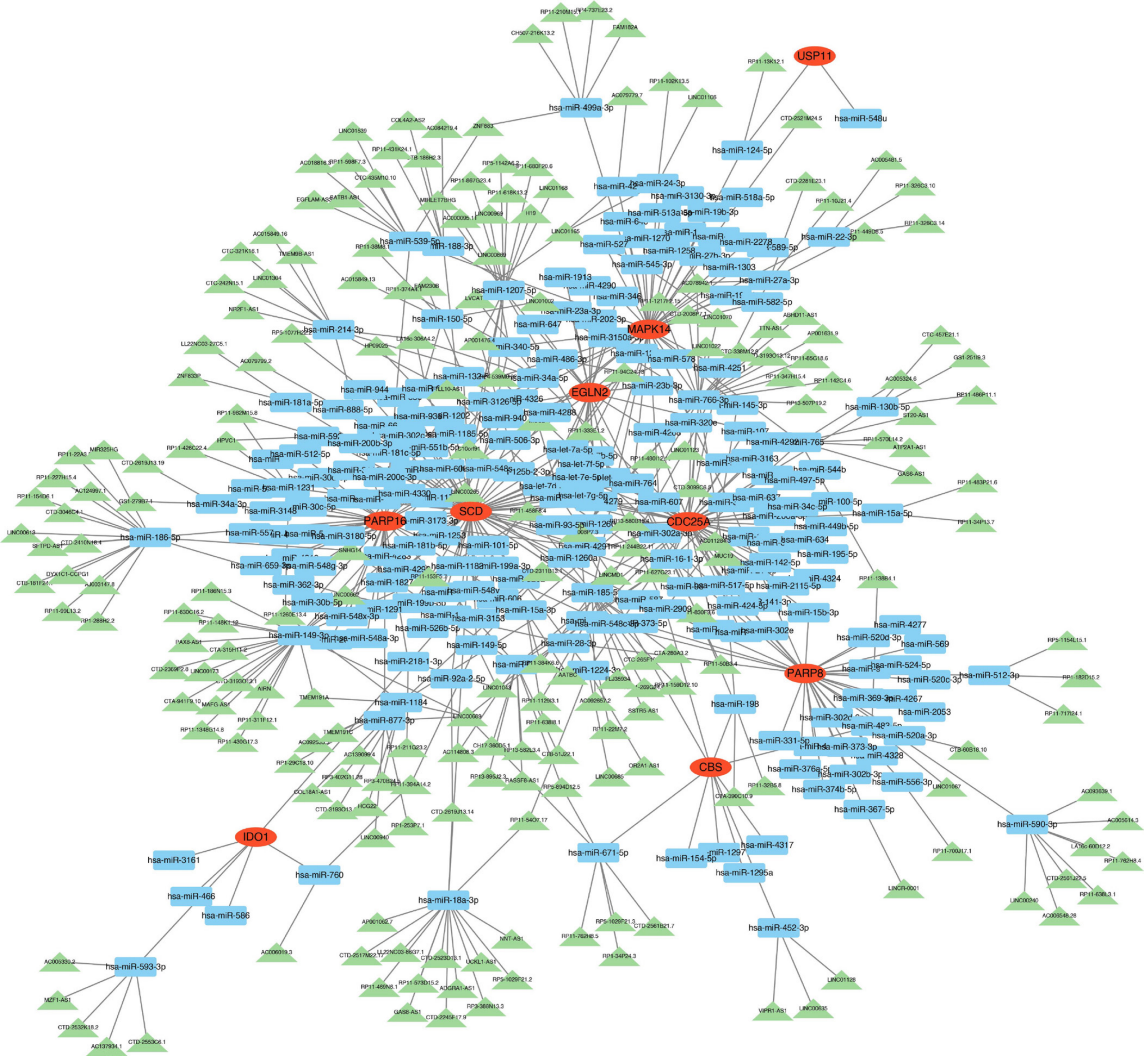
ceRNA networks based on marker genes. The ceRNA network comprised 447 nodes (9 hub DE-FRGs, 217 miRNAs and 221 lncRNAs) and 558 edges, highlighting the complex interactions among these hub genes.

Next, these hub DE-FRGs were used to define a ceRNA network comprising 447 nodes (9 DE-FRGs, 217 miRNAs,= and 221 lncRNAs) and 558 edges (Fig. 9), highlighting the complex interactions among these genes. As an example, 16, 12 and 5 lncRNAs were predicted to control SCD expression by respectively competitively binding hsa-miR-186–5 p, hsa-miR-18a-3p and miR-188–3 p. Moreover, the regulation of PARP16 was putatively controlled by the respective competitive binding of 9, 7 and 3 lncRNAs to hsa-miR-214–3 p, hsa-miR-539–5 p and hsa-miR-877–3 p. The lncRNA RP4-539M6.22 was also predicted to be capable of controlling SCD, MAPK14, PARP8, PARP16 and EGLN2 expression through interactions with hsa-miR-922, hsa-miR-486–3 p, hsa-miR-302a-3p, hsa-miR-766–3 p and hsa-miR-1207–5 p. For further details regarding this ceRNA network, see File S7.

## Discussion

The death of host cells is essential for the appropriate regulation of Mtb infections, preventing mycobacterial growth and spread [8–10]. While apoptotic death is a defensive strategy engaged by cells combatting intracellular pathogens [9], necrotic death is unregulated such that it can allow Mtb to spread to proximal cells upon lytic death [10]. Ferroptotic death shares certain characteristics with both apoptosis and necrosis, and has been reported to promote the growth and dissemination of Mtb, contributing to lethal outcomes in infected mice [12, 13]. Here, a series of systematic bioinformatics analyses were used to explore important FRGs associated with the differentiation of ATB from LTBI in children. The relationship between these key FRGs and immune cell function was also analysed, as were related biological functions in an effort to offer further insight into the potential pathogenic mechanisms that govern ATB onset.

Of the nine hub DE-FRGs identified in this analysis, the three that exhibited the greatest predictive utility when differentiating between paediatric LTBI and ATB samples were *EGLN2*, *MAPK14* and *PARP16*. Mitogen-activated protein kinase 14 (*MAPK14*) is an important driver of ferroptosis such that inhibiting the kinase activity of this protein *in vitro* can disrupt ferroptotic death [15]. Consistent with the present findings, Petrilli *et al*. [16] reported a significant increase in *MAPK14* mRNA levels in ATB patient samples that could reliably distinguish these samples from those of LTBI patients. However, these previous studies did not focus specifically on ferroptosis-related genes and paediatrics, which may be the reason why several genes that were previously observed to distinguish ATB from LTB1 were not identified in our study [17]. Poly (ADP-ribose) polymerases (*PARP*) family members are cytosolic and nuclear proteins that play diverse roles in metabolic regulation, transcriptional control, DNA repair, the maintenance of genomic integrity and DNA methylation, and programmed cell death responses [18]. In ovarian cancer cells, treatment with the PARP inhibitor Olaparib can trigger ferroptotic death through the suppression of light chain subunit solute carrier family 7 member 11 (*SLC7A11*)-mediated GSH biosynthesis [19]. The upregulation of pro-ferroptotic genes such as *MAPK14*, *CBS* and *SCD*, together with the downregulation of *PARP16* and other anti-ferroptotic genes may play a key role in Mtb dissemination over the course of LTBI progression to ATB. The EGLN proline hydroxylase family member *EGLN2* [20] has also been linked to ferroptosis such that *EGLN2* suppression can limit the ability of the small molecule cystine-glutamate exchanger system x or GPX4 inhibitor RSL-3 to induce ferroptosis [21]. Given that ferroptosis entails high levels of inflammation and tissue damage related to the release of intracellular compounds [22], downregulating pro-ferroptotic genes such as *EGLN2*, *USP11* and *IDO1* while upregulating anti-ferroptotic factors like *CDC25A* and *PARP8* may help to limit Mtb spread and associated damage.

The immune response is central to the control and dissemination of Mtb within infected hosts [23]. In line with previous findings [24–28], the analysis of immune cells in the present study showed significant increases in inflammatory and myeloid cell populations such as neutrophils, monocytes and DCs in samples from ATB patients compared with LTB1 samples, whereas B and T cell levels showed the opposite trend. In their prior analyses of samples from ATB patients, Berry *et al*. [24] observed decreases in the expression of B and T cell-specific genes, and were able to confirm that effector and central memory T cell responses were reduced in these patients using a flow cytometry-based validation strategy. This aligns well with recent evidence highlighting a link between the monocyte/lymphocyte ratio and the odds of ATB onset following Mtb infection [25, 26]. Adaptive cellular immune responses are important mediators of the establishment of chronic LTBI infections, with CD4^+^ T cell-derived cytokines serving to enhance CD8^+^ T cell proliferation and the production of antibodies by B cells in a manner that can aid in the control of Mtb-infected macrophages. Indeed, Mtb-reactive MHC-I restricted CD8^+^ T cells can protect against Mtb dissemination in human LTBI patients, while B cells can function in germinal centres to generate antibodies that can coordinate adaptive and innate immune responses by improving antigen presentation to T cells and generating cytokines capable of augmenting ongoing immune responses [23, 27, 28]. Granuloma formation and Mtb infection course can be profoundly shaped by such T cell activity and antibody production [27, 28]. In an integrated analysis of eight distinct TB-focused microarray datasets, Joosten *et al*. [29] determined that TREM1 signalling activity was closely related to the ATB-associated activity of myeloid cells. At a functional level, the expression of TREM1 was able to strengthen monocytic and neutrophil-mediated inflammatory responses. When lymphocytic responses are impaired, this often favours poorer Mtb control such that LTBI can progress to ATB. In ATB patients, bacterial spread and associated tissue damage can drive high levels of persistent inflammatory activity, in turn promoting DC, neutrophil, monocyte and macrophage proliferation. Ferroptotic cell death has been shown to be more immunogenic and pro-inflammatory than apoptotic death, potentially serving as an initiating proinflammatory event [30, 31]. Here, several of the identified hub DE-FRGs were found to be enriched in immune and inflammatory response pathways. Moreover, these FRGs were correlated with inflammatory and lymphocyte cell populations in opposing directions, in line with the above data. However, some genes may play different roles in different sites of infection. For example, *MAPK14* deletion led to increased recruitment of macrophages and neutrophils in mouse CNS injuries [32]. Additionally, these results are merely correlative, and additional research will be required to assess potential causality.

Lastly, candidate drugs with the potential to target these hub genes were identified, and a ceRNA network was established. These drugs included the p38 MAPK inhibitor doramapimod. *In vitro,* p38 MAPK signalling is important for a range of stress-related and inflammatory responses in host cells infected by Mtb [33]. Hölscher *et al*. [34] found that using doramapimod to treat mice infected with Mtb was sufficient to reduce inflammatory activity, lung pathology and granuloma formation without causing any pronounced toxicity. Combining doramapimod and standard antibiotic regimens in these animals also lowered the mycobacterial burden in the spleen and lungs more readily than antibiotic treatment in isolation. Given these findings and the present results, doramapimod represents an attractive tool to protect against LTBI progression to ATB in paediatric populations through the inhibition of ferroptotic activity. Mtb infection can also alter host miRNA expression profiles [35], with such miRNA dysregulation potentially altering the induction of innate immune responses or bacterial replication [36, 37]. While the specific benefits and roles of the candidate drugs and non-coding RNAs identified herein remain to be established, they provide a strong foundation for future research.

This study is subject to multiple limitations. For one, this was a small retrospective analysis, potentially limiting the accuracy of these results. Additionally, the specific focus on subsets of hub genes specifically associated with ferroptosis may have led to our overlooking the relevance of many other important genes associated with the transition between ATB and LTBI during the process of prognostic model construction. Furthermore, while this study did identify multiple FRGs associated with paediatric ATB compared to LTBI, all of these samples were derived from children in Africa and have the potential to not be unique to Mtb infection or to not be generalizable to other ethnic groups. As such, additional *in vitro* and *in vivo* analyses will be vital to fully understand how these FRGs govern the progression of childhood ATB to LTBI.

## Conclusions

In summary, in this study nine hub FRGs were identified as candidate biomarkers for distinguishing between ATB and LTBI in children. Furthermore, they may potentially participate in the pathogenic process governing the transition between ATB and LTBI patients in a paediatric population. These genes thus offer value in diagnosis and treatment of Mtb infection in children.

## Supplementary Data

Supplementary material 1
