# Supplementary material for: Assessment of ferroptosis-associated gene signatures as potential biomarkers for differentiating latent from active tuberculosis in children
Source: Microb Genom. 2023 May 10;9(5):mgen000997. doi: 10.1099/mgen.0.000997 (PMC10272887; doi:10.1099/mgen.0.000997)
Supplement: Supplementary material 1 [file mgen-9-997-s001.pdf]

Supplementary file 1: 728 FRGs identified in this study
PTGS2
CHAC1
SLC40A1
TF
TFRC
FTH1
GPX4
HSPB1
NFE2L2
GPX4
FTH1
RPL8
IREB2
ATP5MC3
CS
EMC2
ACSF2
NOX1
CYBB
NOX3
NOX4
NOX5
DUOX1
DUOX2
G6PD
PGD
VDAC2
PIK3CA
FLT3
SCP2
TP53
ACSL4
LPCAT3
NRAS
KRAS
HRAS
TF
TFRC
TFR2
SLC38A1
SLC1A5
GLS2
GOT1
CARS1
TP53
ALOX5
KEAP1
HMOX1
TP53
TP53

GLS2
ATG5
ATG7
NCOA4
TF
ALOX5
ALOX12
ALOX12B
ALOX15
ALOX15B
ALOXE3
PHKG2
TFRC
ACO1
IREB2
SLC38A1
GLS2
G6PDX
ULK1
ATG3
ATG4D
ATG5
BECN1
MAP1LC3A
GABARAPL2
GABARAPL1
ATG16L1
WIPI1
WIPI2
SNX4
ATG13
ULK2
NCOA4
ACSL4
TP53
SAT1
ALOX15
ACSL4
LPCAT3
ALOX15
ACSL4
KEAP1
EGFR
NOX4
MAPK3
MAPK1
BID
ACSL4
ZEB1
KEAP1
DPP4

ALOX15
ALOX12
CDKN2A
PEBP1
SOCS1
CDO1
MYB
HMOX1
MAPK8
MAPK9
MAPK1
MAPK3
SLC1A5
CHAC1
MAPK14
LINC00472
NOX4
GOT1
BECN1
PRKAA2
PRKAA1
ELAVL1
BAP1
TP53
ABCC1
ACSL4
MIR6852
ACVR1B
TGFBR1
BAP1
EPAS1
HILPDA
HIF1A
ALOX12
ACSL4
HMOX1
IFNG
ANO6
LPIN1
HMGB1
TNFAIP3
TLR4
NOX4
ATF3
ATM
YY1AP1
EGLN2
MIOX
TAFAZZIN
MTDH
IDH1

SIRT1
TAFAZZIN
BECN1
FBXW7
PANX1
DNAJB6
BACH1
ACSL4
LONP1
CD82
IL1B
CTSB
POR
CYB5R1
ELOVL5
FADS1
ALOX12
FBXW7
PTEN
NR1D1
NR1D2
TBK1
IL6
USP7
miR-182-5p
miR-378a-3p
CTSB
ACSL4
ATF4
BECN1
AQP3
AQP5
AQP8
LINC00618
IREB2
MT1DP
ACSL4
PEX10
KEAP1
AGPAT3
PEX12
CHP1
GPAT4
BRPF1
OSBPL9
INTS2
MMD
CYP4F8
MLLT1
TTPA
GRIA3

EPT1
POM121L12
LIG3
AEBP2
AGPS
CDCA3
PEX2
LPCAT3
PEX6
TIMM9
DCAF7
LCE2C
FAR1
PHF21A
SMAD7
LYRM1
AMN
PEX3
MTCH1
ZEB1
SIRT1
ACADSB
PVT1
hsa_circ_0008367
SLC39A14
NCOA4
MAP3K11
GSK3B
MAPK8
BRD7
TP53
SLC25A28
ACSL4
MFN2
ACSL4
SLC11A2
ZFAS1
SLC38A1
TSC1
PEBP1
TGFB1
SNCA
SIRT3
PRKAA2
TFRC
CGAS
STING1
HDDC3
MIR761
MDM2
MDM4

ALOX15
POR
MIR214
DLD
LONP1
ACSL4
BACH1
DNAJB6
WWTR1
SIRT1
ATM
PRKCA
LGMN
ACSL4
TP53
IFNG
SMPD1
MYCN
SLC11A2
IFNA1
IFNA2
IFNA4
IFNA5
IFNA6
IFNA7
IFNA8
IFNA10
IFNA13
IFNA14
IFNA16
IFNA17
IFNA21
SMG9
NR1D1
ACSL4
PPARG
TLR4
IL6
MIR335
ATF3
HMOX1
HMGB1
EPAS1
SNX5
PAQR3
MICU1
NOX4
TOR2A
MIR375
MAP3K14
SIRT3

CircKDM4C
MIR324
QSOX1
MIB2
CLTRN
KLF2
MIR5096
TFRC
HOTAIR
H19
FOXO4
ELAVL1
YTHDC2
DDR2
SLC39A7
TRIM46
ACSL1
KDM5A
TRIM21
HMOX1
DPEP1
CYGB
IDO1
GSTZ1
TP53
ACO1
GJA1
IREB2
SLC7A11
PGRMC1
CIRBP
FAR1
circPSEN1
USP11
STING1
YAP1
HMOX1
MIR135B
TRIM26
YAP1
NDRG1
MIR302A
ASMTL-AS1
ZFAS1
FADS2
PIEZO1
LIFR
PTPN6
MIR15A
EGR1
ADAM23

ARHGEF26-AS1
ACSL4
CPEB1
COX4I2
lncRNA AABR07017145.1
TIMP1
MIR15A
KDM6B
NCOA4
GSK3B
IFNG
METTL14
CHAC1
MIB1
KDM5C
ACSL4
MEG3
CCDC6
ATF3
IREB2
CFL1
ALOXE3
MIR539
KMT2D
SLC7A11
GPX4
AKR1C1
AKR1C2
AKR1C3
GPX4
RB1
HSPB1
HSF1
SLC7A11
GPX4
GCLC
SLC7A11
NFE2L2
SQSTM1
NQO1
HMOX1
FTH1
MUC1
SLC3A2
MT1G
NFE2L2
SLC40A1
SLC7A11
GPX4
SLC7A11
CISD1

SLC7A11
FANCD2
GPX4
NFE2L2
FTMT
HSPA5
ATF4
SLC7A11
GPX4
GPX4
HMOX1
ATF4
NFE2L2
TP53
SLC7A11
HELLS
SCD
FADS2
SRC
STAT3
NFE2L2
PML
MTOR
NFS1
TP63
SLC7A11
TP53
CDKN1A
MIR137
SLC40A1
GPX4
GPX4
ENPP2
VDAC2
FH
CISD2
SLC40A1
MIR9-1
MIR9-2
MIR9-3
CBS
NFE2L2
SQSTM1
GPX4
ISCU
FTH1
ACSL3
OTUB1
CD44
LINC00336
STAT3

BRD4
PRDX6
MIR17
SCD
SESN2
NF2
ARNTL
HIF1A
JUN
CA9
HSPA5
TMBIM4
HSPA5
PLIN2
MIR212
Fer1HCH
AIFM2
AIFM2
LAMP2
ZFP36
GPX4
PROM2
CHMP5
CHMP6
AKR1C1
AKR1C2
AKR1C3
CBS
NFE2L2
CAV1
GCH1
SIRT3
DAZAP1
PIR
GCLC
FTL
HCAR1
SLC16A1
RRM2
SCD
NR4A1
PIK3CA
RPTOR
SREBF1
SREBF2
FZD7
NFE2L2
NFE2L2
P4HB
NT5DC2
BCAT2

HSF1
PLA2G6
MIR424
PARK7
FXN
SUV39H1
ATF2
CDKN1A
FTH1
NFE2L2
STAT3
ACOT1
NFE2L2
ALDH3A2
NFE2L2
STK11
FNDC5
CircIL4R
CDH1
NFE2L2
MIR214
NEDD4L
SQSTM1
TF
FTMT
BRD2
BRD3
BRD4
BRDT
SCD
SLC7A11
DECR1
NFE2L2
GPX4
SLC7A11
NFE2L2
GLRX5
GPX4
NCOA3
NR5A2
GPX4
MTOR
PANX2
RHEBP1
TFAP2A
CP
SLC7A11
ARF6
GDF15
ABHD12
PPP1R13L

TFAM
KDM3B
RNF113A
PARK7
AHCY
FXN
circ-TTBK2
MIR522
IDH2
PPARA
NOS2
SIAH2
RELA
PRKAA2
VDR
NEDD4
FXN
AIFM2
PRDX1
AR
CBS
NFE2L2
CHMP5
CHMP6
HMOX1
ZFP36
LAMP2
MTF1
COPZ1
NUPR1
USP35
HSF1
PROM2
PLA2G6
HIF1A
NEAT1
RRM2
SLC7A11
FTMT
PARP1
PARP2
PARP3
PARP4
PARP6
PARP8
PARP9
PARP10
PARP11
PARP12
PARP14
PARP15

PARP16
PDSS2
TXN
SENP1
PLA2G6
OIP5-AS1
MIR190A
FGF21
CREB1
CREB3
CREB5
FTMT
GOT1
TFRC
GPX4
MIR130B
BEX1
ASAH2
SCD
FABP4
AKT1S1
MLST8
MTOR
RPTOR
CDH1
SIRT1
TYRO3
SIRT6
TMSB4X
TMSB4Y
KIF20A
ECH1
circRHOT1
ETV4
MEG8
VCP
circ_0007142
ENPP2
RBMS1
KDM4A
CBS
MGST1
circKIF4A
miR-7-5p
PRDX6
circ_0067934
MPC1
CHMP1A
CAMKK2
SOX2
SRSF9

PROK2
MIR4443
SIRT2
circRNA1615
MIR27A
MIR670
MEF2C
NF2
CDH1
HSPB1
EZH2
PEDS1
SMPD1
ADAMTS13
CDC25A
G6PD
SRSF9
CAV1
CircFNDC3B
PPARD
CISD2
ENO3
SESN2
LCN2
MARCHF5
TRIB2
DHODH
SLC7A11
MIR545
OTUB1
PDK4
CircPVT1
MIR9-3HG
ADIPOQ
circDTL
GPX4
mmu_circRNA_0000309
IL6
PTPN18
FTH1
FTH1
FTL
LCN2
ABCC5
CISD3
MS4A15
LCN2
FURIN
circRHBG
GALNT14
KLHDC3

LINC01833
circGFRA1
MAPKAP1
MLST8
MTOR
PRR5
RICTOR
GSTM1
TERT
circ0097009
TMEM161B-DT
circEPSTI1
MIR18A
RARRES2
USP11

Supplementary file 2: DE-FRGs identified in this study

Gene	conMean	treatMean	pvalue	Type
FTH1	9.661171	10.41003	0.000646	Up
HSPB1	11.84735	12.27082	0.045516	Up
NFE2L2	10.28948	10.67486	0.001104	Up
CYBB	10.49719	10.98702	0.000646	Up
ACSL4	7.409579	7.988181	0.003619	Up
NRAS	7.379683	7.619157	0.01182	Up
SLC1A5	8.328388	9.18162	0.007488	Up
ATG7	9.246197	9.64786	0.010808	Up
NCOA4	14.49482	14.895	0.008604	Up
ALOX15B	3.573464	4.283485	0.006816	Up
ATG3	10.04015	10.39994	0.016055	Up
GABARAPL2	12.12092	12.63736	0.007846	Up
WIPI2	7.640926	8.085075	0.039151	Up
SAT1	13.5861	13.91438	0.022476	Up
MAPK1	10.20241	10.40173	0.028095	Up
SOCS1	8.034726	8.535723	0.021569	Up
MAPK14	7.209724	8.180239	7.99E-05	Up
TNFAIP3	9.49767	9.731151	0.027541	Up
TLR4	8.584235	9.108361	0.020692	Up
ATF3	3.596508	4.573374	0.029731	Up
IDH1	8.766906	9.152259	0.000812	Up
BACH1	5.715302	6.291205	0.00499	Up
CD82	9.277436	9.532628	0.031029	Up
IL1B	10.0292	10.72191	0.004415	Up
CTSB	12.18838	12.57021	0.0065	Up
FADS1	5.738481	6.472331	0.008608	Up
TBK1	9.721368	10.06696	0.009874	Up
AGPAT3	6.154051	6.631492	0.009013	Up
AGPS	8.531385	8.700456	0.031029	Up
PHF21A	12.08787	12.37198	0.009434	Up
LYRM1	11.01533	11.24396	0.041435	Up
PPARG	0	0.91744	0.020013	Up
TOR2A	6.064195	7.155685	0.002283	Up
QSOX1	8.840373	9.250022	0.00591	Up
ACSL1	10.6617	11.45738	0.018245	Up
TRIM21	10.35881	10.70193	0.025409	Up
EGR1	7.515309	7.925327	0.016758	Up
TIMP1	12.53124	12.84534	0.036263	Up
RB1	7.889328	8.090153	0.033559	Up
GCLC	6.810493	7.590243	0.006199	Up
MUC1	4.360129	5.299217	0.006199	Up
MT1G	5.289178	6.678372	0.002055	Up
SCD	8.035338	8.318074	0.042233	Up
STAT3	10.49295	10.78745	0.013496	Up
PML	6.205857	6.573427	0.036263	Up
CISD2	8.541556	9.361827	0.014099	Up
CBS	3.867826	5.72294	0.000266	Up
LAMP2	11.04398	11.34607	0.00922	Up
CHMP5	9.634084	10.12682	0.045516	Up

GCH1	8.193039	8.61361	0.012357	Up
PIR	3.718951	4.353557	0.010331	Up
FTL	15.539	15.721	0.00039	Up
GLRX5	14.33194	14.66817	0.035565	Up
SIAH2	10.84373	11.61346	0.022479	Up
VDR	4.726917	5.373059	0.000235	Up
MTF1	10.27493	10.73562	0.002956	Up
PARP8	7.732365	8.186997	0.001074	Up
PARP9	10.76016	11.35413	0.003998	Up
PARP14	10.73175	11.07102	0.031029	Up
TXN	11.85391	12.40251	0.002808	Up
CREB5	8.525543	9.461835	0.001016	Up
BEX1	5.331706	5.968079	0.013496	Up
MGST1	5.865392	6.769737	0.002283	Up
CDC25A	2.430809	4.004714	0.01657	Up
LCN2	9.293172	10.61773	0.000483	Up
FURIN	8.347847	9.023222	0.045514	Up
GALNT14	2.658784	4.211153	0.008329	Up
RPL8	13.60477	13.39631	0.037676	Down
CS	11.31625	10.95026	0.000178	Down
ACSF2	6.179426	5.171913	0.008607	Down
SLC38A1	11.54272	11.08641	0.004415	Down
GOT1	9.314546	9.052727	0.033559	Down
ALOX15	6.711358	5.442239	4.10E-05	Down
PHKG2	8.103097	7.679196	0.002002	Down
ATG16L1	10.45278	10.10294	0.000114	Down
MYB	9.024325	8.538985	0.0012	Down
ELAVL1	7.545144	7.178121	0.022479	Down
LPIN1	11.07584	10.65077	0.000745	Down
ATM	10.66184	10.16679	0.00211	Down
EGLN2	11.76718	11.49284	6.34E-05	Down
NR1D2	7.65487	7.120609	0.013496	Down
BRPF1	10.90563	10.64336	0.000724	Down
OSBPL9	8.969591	8.838102	0.04384	Down
DCAF7	11.37533	11.12974	0.002166	Down
SMAD7	6.984349	6.601754	0.012916	Down
MTCH1	12.3833	12.27818	0.027528	Down
MYCN	3.030152	1.930638	0.045892	Down
PAQR3	6.97876	6.038247	0.014726	Down
MAP3K14	7.598127	7.228148	0.003272	Down
MIB2	8.152308	7.772945	0.001492	Down
TRIM46	5.559273	4.769634	0.00311	Down
IDO1	9.304929	7.688897	0.002667	Down
CIRBP	12.11743	11.76135	0.004637	Down
USP11	7.701772	7.438748	0.034887	Down
MIR15A	2.17575	1.01487	0.020115	Down
ADAM23	4.446978	2.049586	0.004268	Down
METTL14	7.653797	7.470665	0.049013	Down
MEG3	3.526506	2.233007	0.00841	Down
CCDC6	11.12956	10.94113	0.029823	Down
AKR1C3	9.125695	8.377995	0.049013	Down

FANCD2	7.216201	6.745669	0.010808	Down
TP63	4.772845	4.144865	0.034881	Down
ENPP2	5.68922	4.31978	0.009393	Down
CD44	12.42414	12.34377	0.040657	Down
SESN2	5.477375	5.021005	0.045516	Down
AIFM2	5.544692	4.743947	0.000575	Down
FXN	4.976668	4.078024	0.042211	Down
SUV39H1	8.810041	8.345532	0.010332	Down
ATF2	7.377156	7.001434	0.049013	Down
ACOT1	4.984156	4.675945	0.045516	Down
ALDH3A2	8.212092	7.844889	0.000183	Down
FNDC5	2.287069	1.111384	0.022043	Down
BRD2	12.64368	12.30773	0.0001	Down
TFAM	10.08128	9.823319	0.014098	Down
PPARA	3.685001	2.150957	0.014614	Down
COPZ1	10.53736	10.35225	0.027541	Down
PARP1	11.9002	11.52642	0.008608	Down
PARP4	11.97741	11.88756	0.043846	Down
PARP15	8.382709	7.88048	0.046368	Down
PARP16	7.758502	7.292124	0.000172	Down
PDSS2	8.513764	8.302037	0.01182	Down
ECH1	11.81685	11.52289	0.001135	Down
TRIB2	10.13505	9.447359	0.000961	Down
KLHDC3	11.80506	11.44287	0.000118	Down

Supplementary file 3: The CIBERSORT algorithm results

Cell	pvalue
Activatec	0.024397
Activatec	0.002283
Activatec	0.000266
Gamma.del	0.001074
MDSC	0.015378
Neutrophi	0.027541
Regulator	0.014099
Effector.	2.44E-06
Central.n	1.17E-05
Central.n	4.39E-05
Effector.	0.000134

Supplementary file 4: prediction of miRNA target genes

Gene	miRNA	miRanda	miRDB	TargetSca	Sum
SCD	hsa-miR-1	1	1	1	3
MAPK14	hsa-miR-5	1	1	1	3
PARP8	hsa-miR-3	1	1	1	3
CDC25A	hsa-miR-4	1	1	1	3
CDC25A	hsa-miR-4	1	1	1	3
SCD	hsa-miR-3	1	1	1	3
CBS	hsa-miR-1	1	1	1	3
CDC25A	hsa-miR-3	1	1	1	3
SCD	hsa-miR-9	1	1	1	3
MAPK14	hsa-miR-3	1	1	1	3
PARP16	hsa-miR-5	1	1	1	3
SCD	hsa-miR-6	1	1	1	3
PARP8	hsa-miR-3	1	1	1	3
EGLN2	hsa-miR-1	1	1	1	3
SCD	hsa-miR-6	1	1	1	3
PARP16	hsa-miR-5	1	1	1	3
EGLN2	hsa-let-7	1	1	1	3
MAPK14	hsa-miR-1	1	1	1	3
SCD	hsa-miR-1	1	1	1	3
SCD	hsa-miR-6	1	1	1	3
SCD	hsa-miR-1	1	1	1	3
PARP16	hsa-miR-5	1	1	1	3
EGLN2	hsa-let-7	1	1	1	3
MAPK14	hsa-miR-2	1	1	1	3
SCD	hsa-miR-2	1	1	1	3
CDC25A	hsa-miR-1	1	1	1	3
CDC25A	hsa-miR-6	1	1	1	3
CDC25A	hsa-miR-1	1	1	1	3
CDC25A	hsa-miR-4	1	1	1	3
CDC25A	hsa-miR-7	1	1	1	3
SCD	hsa-miR-5	1	1	1	3
PARP8	hsa-miR-5	1	1	1	3
MAPK14	hsa-miR-3	1	1	1	3
MAPK14	hsa-miR-2	1	1	1	3
MAPK14	hsa-miR-1	1	1	1	3
MAPK14	hsa-miR-1	1	1	1	3
PARP8	hsa-miR-4	1	1	1	3
SCD	hsa-miR-5	1	1	1	3
PARP8	hsa-miR-4	1	1	1	3
EGLN2	hsa-miR-1	1	1	1	3
EGLN2	hsa-miR-5	1	1	1	3
SCD	hsa-miR-1	1	1	1	3
CDC25A	hsa-miR-4	1	1	1	3
CBS	hsa-miR-4	1	1	1	3
CBS	hsa-miR-4	1	1	1	3
SCD	hsa-miR-1	1	1	1	3
MAPK14	hsa-miR-4	1	1	1	3
SCD	hsa-miR-2	1	1	1	3
PARP8	hsa-miR-3	1	1	1	3

EGLN2	hsa-miR-3	1	1	1	3
CDC25A	hsa-let-7	1	1	1	3
PARP16	hsa-miR-1	1	1	1	3
SCD	hsa-miR-4	1	1	1	3
CDC25A	hsa-miR-4	1	1	1	3
CDC25A	hsa-miR-1	1	1	1	3
SCD	hsa-let-7	1	1	1	3
CDC25A	hsa-miR-4	1	1	1	3
CDC25A	hsa-miR-4	1	1	1	3
PARP8	hsa-miR-3	1	1	1	3
MAPK14	hsa-miR-4	1	1	1	3
SCD	hsa-miR-2	1	1	1	3
MAPK14	hsa-miR-7	1	1	1	3
PARP16	hsa-miR-1	1	1	1	3
CDC25A	hsa-miR-1	1	1	1	3
CDC25A	hsa-miR-1	1	1	1	3
PARP8	hsa-miR-3	1	1	1	3
CDC25A	hsa-let-7	1	1	1	3
MAPK14	hsa-miR-4	1	1	1	3
PARP16	hsa-miR-6	1	1	1	3
CDC25A	hsa-miR-1	1	1	1	3
MAPK14	hsa-miR-1	1	1	1	3
SCD	hsa-miR-1	1	1	1	3
CBS	hsa-miR-1	1	1	1	3
MAPK14	hsa-miR-4	1	1	1	3
SCD	hsa-miR-3	1	1	1	3
PARP16	hsa-miR-3	1	1	1	3
PARP8	hsa-miR-3	1	1	1	3
CBS	hsa-miR-1	1	1	1	3
PARP16	hsa-miR-5	1	1	1	3
MAPK14	hsa-miR-7	1	1	1	3
EGLN2	hsa-let-7	1	1	1	3
SCD	hsa-miR-1	1	1	1	3
SCD	hsa-miR-5	1	1	1	3
PARP16	hsa-miR-3	1	1	1	3
PARP16	hsa-miR-3	1	1	1	3
MAPK14	hsa-miR-3	1	1	1	3
SCD	hsa-miR-4	1	1	1	3
CDC25A	hsa-miR-3	1	1	1	3
MAPK14	hsa-miR-5	1	1	1	3
PARP8	hsa-miR-3	1	1	1	3
CDC25A	hsa-miR-4	1	1	1	3
SCD	hsa-miR-1	1	1	1	3
CBS	hsa-miR-6	1	1	1	3
SCD	hsa-miR-4	1	1	1	3
PARP16	hsa-miR-2	1	1	1	3
PARP8	hsa-miR-5	1	1	1	3
SCD	hsa-miR-1	1	1	1	3
PARP16	hsa-miR-5	1	1	1	3
CDC25A	hsa-miR-1	1	1	1	3
PARP8	hsa-miR-5	1	1	1	3

USP11	hsa-miR-1	1	1	1	3
CDC25A	hsa-let-7	1	1	1	3
SCD	hsa-let-7	1	1	1	3
PARP16	hsa-miR-5	1	1	1	3
PARP16	hsa-miR-2	1	1	1	3
SCD	hsa-miR-3	1	1	1	3
PARP16	hsa-miR-6	1	1	1	3
CDC25A	hsa-miR-6	1	1	1	3
EGLN2	hsa-let-7	1	1	1	3
PARP8	hsa-miR-6	1	1	1	3
PARP16	hsa-miR-3	1	1	1	3
EGLN2	hsa-miR-4	1	1	1	3
EGLN2	hsa-let-7	1	1	1	3
CDC25A	hsa-miR-1	1	1	1	3
MAPK14	hsa-miR-1	1	1	1	3
SCD	hsa-miR-4	1	1	1	3
PARP16	hsa-miR-7	1	1	1	3
PARP16	hsa-miR-5	1	1	1	3
CDC25A	hsa-let-7	1	1	1	3
PARP8	hsa-miR-4	1	1	1	3
PARP16	hsa-miR-4	1	1	1	3
CDC25A	hsa-miR-2	1	1	1	3
PARP8	hsa-miR-1	1	1	1	3
PARP16	hsa-miR-3	1	1	1	3
PARP8	hsa-miR-9	1	1	1	3
PARP8	hsa-miR-5	1	1	1	3
PARP16	hsa-miR-4	1	1	1	3
PARP8	hsa-miR-3	1	1	1	3
CDC25A	hsa-miR-1	1	1	1	3
SCD	hsa-miR-3	1	1	1	3
SCD	hsa-let-7	1	1	1	3
CDC25A	hsa-miR-1	1	1	1	3
SCD	hsa-miR-1	1	1	1	3
EGLN2	hsa-miR-9	1	1	1	3
SCD	hsa-miR-6	1	1	1	3
SCD	hsa-miR-4	1	1	1	3
SCD	hsa-miR-3	1	1	1	3
SCD	hsa-miR-5	1	1	1	3
PARP16	hsa-miR-5	1	1	1	3
CDC25A	hsa-miR-3	1	1	1	3
SCD	hsa-miR-1	1	1	1	3
SCD	hsa-miR-1	1	1	1	3
SCD	hsa-let-7	1	1	1	3
EGLN2	hsa-miR-9	1	1	1	3
MAPK14	hsa-miR-1	1	1	1	3
PARP16	hsa-miR-1	1	1	1	3
CDC25A	hsa-miR-1	1	1	1	3
PARP8	hsa-miR-2	1	1	1	3
MAPK14	hsa-miR-4	1	1	1	3
MAPK14	hsa-miR-1	1	1	1	3
MAPK14	hsa-miR-2	1	1	1	3

CDC25A	hsa-miR-5	1	1	1	3
SCD	hsa-miR-9	1	1	1	3
PARP16	hsa-miR-5	1	1	1	3
USP11	hsa-miR-5	1	1	1	3
SCD	hsa-miR-1	1	1	1	3
SCD	hsa-miR-1	1	1	1	3
SCD	hsa-miR-9	1	1	1	3
EGLN2	hsa-miR-3	1	1	1	3
PARP8	hsa-miR-5	1	1	1	3
MAPK14	hsa-miR-3	1	1	1	3
MAPK14	hsa-miR-2	1	1	1	3
EGLN2	hsa-miR-2	1	1	1	3
EGLN2	hsa-miR-2	1	1	1	3
SCD	hsa-miR-3	1	1	1	3
MAPK14	hsa-miR-7	1	1	1	3
IDO1	hsa-miR-5	1	1	1	3
MAPK14	hsa-miR-3	1	1	1	3
CDC25A	hsa-miR-3	1	1	1	3
SCD	hsa-miR-3	1	1	1	3
SCD	hsa-miR-1	1	1	1	3
SCD	hsa-miR-8	1	1	1	3
CBS	hsa-miR-1	1	1	1	3
CBS	hsa-miR-2	1	1	1	3
CDC25A	hsa-let-7	1	1	1	3
MAPK14	hsa-miR-5	1	1	1	3
CDC25A	hsa-miR-4	1	1	1	3
PARP8	hsa-miR-4	1	1	1	3
SCD	hsa-miR-1	1	1	1	3
IDO1	hsa-miR-4	1	1	1	3
PARP16	hsa-miR-1	1	1	1	3
CDC25A	hsa-miR-1	1	1	1	3
EGLN2	hsa-miR-4	1	1	1	3
IDO1	hsa-miR-1	1	1	1	3
PARP16	hsa-miR-3	1	1	1	3
SCD	hsa-miR-1	1	1	1	3
CDC25A	hsa-miR-3	1	1	1	3
SCD	hsa-miR-1	1	1	1	3
MAPK14	hsa-miR-5	1	1	1	3
MAPK14	hsa-miR-1	1	1	1	3
MAPK14	hsa-miR-5	1	1	1	3
SCD	hsa-miR-5	1	1	1	3
CBS	hsa-miR-4	1	1	1	3
PARP8	hsa-miR-2	1	1	1	3
CDC25A	hsa-miR-1	1	1	1	3
CDC25A	hsa-miR-6	1	1	1	3
SCD	hsa-miR-6	1	1	1	3
PARP16	hsa-miR-2	1	1	1	3
SCD	hsa-miR-7	1	1	1	3
SCD	hsa-miR-5	1	1	1	3
MAPK14	hsa-miR-5	1	1	1	3
MAPK14	hsa-miR-3	1	1	1	3

SCD	hsa-miR-9	1	1	1	3
MAPK14	hsa-miR-4	1	1	1	3
MAPK14	hsa-miR-2	1	1	1	3
CDC25A	hsa-miR-2	1	1	1	3
SCD	hsa-miR-1	1	1	1	3
SCD	hsa-miR-4	1	1	1	3
PARP16	hsa-miR-3	1	1	1	3
EGLN2	hsa-let-7	1	1	1	3
SCD	hsa-miR-1	1	1	1	3
CDC25A	hsa-miR-2	1	1	1	3
PARP8	hsa-miR-3	1	1	1	3
CDC25A	hsa-miR-1	1	1	1	3
PARP16	hsa-miR-4	1	1	1	3
MAPK14	hsa-miR-4	1	1	1	3
MAPK14	hsa-miR-5	1	1	1	3
PARP8	hsa-miR-5	1	1	1	3
SCD	hsa-let-7	1	1	1	3
PARP8	hsa-miR-5	1	1	1	3
CDC25A	hsa-miR-3	1	1	1	3
MAPK14	hsa-miR-6	1	1	1	3
SCD	hsa-miR-9	1	1	1	3
SCD	hsa-miR-9	1	1	1	3
PARP8	hsa-miR-5	1	1	1	3
SCD	hsa-miR-1	1	1	1	3
SCD	hsa-let-7	1	1	1	3
CDC25A	hsa-miR-9	1	1	1	3
SCD	hsa-miR-5	1	1	1	3
MAPK14	hsa-miR-1	1	1	1	3
PARP8	hsa-miR-3	1	1	1	3
SCD	hsa-miR-3	1	1	1	3
CDC25A	hsa-miR-1	1	1	1	3
SCD	hsa-miR-1	1	1	1	3
CBS	hsa-miR-4	1	1	1	3
CDC25A	hsa-let-7	1	1	1	3
EGLN2	hsa-miR-2	1	1	1	3
EGLN2	hsa-miR-1	1	1	1	3
EGLN2	hsa-let-7	1	1	1	3
MAPK14	hsa-miR-3	1	1	1	3
EGLN2	hsa-miR-6	1	1	1	3
IDO1	hsa-miR-5	1	1	1	3
PARP16	hsa-miR-5	1	1	1	3
SCD	hsa-miR-1	1	1	1	3
CDC25A	hsa-miR-9	1	1	1	3
EGLN2	hsa-miR-4	1	1	1	3
CDC25A	hsa-miR-3	1	1	1	3
PARP8	hsa-miR-5	1	1	1	3
CDC25A	hsa-miR-5	1	1	1	3
IDO1	hsa-miR-3	1	1	1	3
CDC25A	hsa-miR-1	1	1	1	3
IDO1	hsa-miR-7	1	1	1	3
MAPK14	hsa-miR-5	1	1	1	3

EGLN2	hsa-miR-5	1	1	1	3
PARP16	hsa-miR-5	1	1	1	3
MAPK14	hsa-miR-6	1	1	1	3
EGLN2	hsa-miR-1	1	1	1	3
EGLN2	hsa-miR-4	1	1	1	3
EGLN2	hsa-miR-7	1	1	1	3
CDC25A	hsa-let-7	1	1	1	3
PARP8	hsa-miR-5	1	1	1	3
PARP8	hsa-miR-3	1	1	1	3
PARP8	hsa-miR-1	1	1	1	3
SCD	hsa-miR-6	1	1	1	3
MAPK14	hsa-miR-1	1	1	1	3
SCD	hsa-miR-1	1	1	1	3
PARP16	hsa-miR-8	1	1	1	3
SCD	hsa-miR-4	1	1	1	3
PARP8	hsa-miR-3	1	1	1	3
PARP16	hsa-miR-4	1	1	1	3
CDC25A	hsa-miR-5	1	1	1	3
SCD	hsa-let-7	1	1	1	3

Supplementary file 5: mRNA-lncRNA interactions

miRNA	lncRNA
hsa-miR-1	LINC01043
hsa-miR-7	GAS6-AS1
hsa-miR-1	HP09025
hsa-miR-2	LA16c-306A4.2
hsa-miR-1	RP11-830F9.6
hsa-miR-2	RP11-10J21.4
hsa-miR-6	RP5-894D12.5
hsa-miR-6	RP5-894D12.5
hsa-miR-6	RP1-34P24.3
hsa-miR-3	C10orf91
hsa-miR-1	C10orf91
hsa-miR-1	MUC19
hsa-miR-4	AC079779.7
hsa-miR-1	LINC01043
hsa-miR-2	RP11-102K13.5
hsa-miR-5	LINC01070
hsa-miR-5	RP11-982M15.8
hsa-miR-4	C10orf91
hsa-miR-6	RP11-627G23.1
hsa-miR-1	CTC-265F19.1
hsa-miR-7	LINC01070
hsa-miR-7	RP11-138B4.1
hsa-miR-1	RP11-982M15.8
hsa-miR-1	AC092657.2
hsa-miR-1	RP11-99L13.2
hsa-miR-1	CTB-186H2.3
hsa-miR-1	TMEM191C
hsa-miR-2	RP11-326C3.10
hsa-miR-2	LINC01106
hsa-miR-1	RP11-830F9.6
hsa-miR-1	RP3-388N13.3
hsa-miR-4	RP4-737E23.2
hsa-miR-4	RP11-138B4.1
hsa-miR-1	RP11-333E1.2
hsa-miR-2	RP4-539M6.22
hsa-miR-1	AC011284.3
hsa-miR-1	AATBC
hsa-miR-7	RP11-627G23.1
hsa-miR-7	RP13-507P19.2
hsa-miR-3	LA16c-306A4.2
hsa-miR-1	LINC01002
hsa-miR-1	LINC01002
hsa-miR-3	AP001476.4
hsa-miR-1	MIR325HG
hsa-miR-1	C10orf91
hsa-miR-1	COL4A2-AS2
hsa-miR-1	LINC00689
hsa-miR-1	RP11-618K13.2
hsa-miR-1	GAS8-AS1

hsa-miR-5RP11-717I24.1
hsa-miR-1RP11-5407.17
hsa-miR-7MUC19
hsa-miR-8LINC00689
hsa-miR-2RP11-326C3.14
hsa-miR-5HPVC1
hsa-miR-1CTA-941F9.10
hsa-miR-4VIPR1-AS1
hsa-miR-1RP11-394A14.2
hsa-miR-7CTD-2008P7.3
hsa-miR-3MUC2
hsa-miR-1LINC00265
hsa-miR-1RP11-333E1.2
hsa-miR-7RP13-580B18.4
hsa-miR-1LINC00685
hsa-miR-1LINC01165
hsa-miR-1RP11-32B5.8
hsa-miR-1AIRN
hsa-miR-1RP3-470B24.5
hsa-miR-1AC084219.4
hsa-miR-7AC078942.1
hsa-miR-4RP11-210M15.1
hsa-miR-1FAM230B
hsa-miR-6CTB-51J22.1
hsa-miR-3RP4-539M6.22
hsa-let-7RP11-94C24.13
hsa-miR-4RP11-94C24.13
hsa-miR-1AP001476.4
hsa-miR-1RP4-539M6.22
hsa-miR-1LL22NC03-27C5.1
hsa-miR-1CTD-2619J13.14
hsa-miR-1RP11-384K6.6
hsa-miR-4FAM182A
hsa-miR-1HCG22
hsa-miR-6CTD-2561B21.7
hsa-miR-4LINC01165
hsa-miR-5RP1-182D15.2
hsa-miR-1FLJ35934
hsa-miR-1HPVC1
hsa-miR-5LINC01165
hsa-miR-1LINC00265
hsa-miR-1LINC01123
hsa-miR-1RP11-311F12.1
hsa-miR-5CTD-2532K18.2
hsa-miR-1RP11-426C22.4
hsa-miR-7RP11-1217F2.15
hsa-miR-4CH507-216K13.2
hsa-miR-1NNT-AS1
hsa-miR-1GS1-251I9.3
hsa-miR-7CTD-3193O13.12
hsa-miR-1LINC00689

hsa-miR-8LINC00940
hsa-miR-1CTD-3099C6.5
hsa-miR-1LINC00613
hsa-miR-2LINC01043
hsa-miR-6RP5-1029F21.3
hsa-miR-1CTD-2517M22.17
hsa-miR-1RP11-269G24.6
hsa-miR-6RP11-762H8.5
hsa-miR-2RP11-449D8.5
hsa-miR-1RP11-1260E13.4
hsa-miR-1RP11-1260E13.4
hsa-miR-2RP11-333E1.2
hsa-miR-1CTD-2008P7.3
hsa-miR-7RP11-85G18.6
hsa-miR-5AC018816.3
hsa-miR-5AC005330.2
hsa-miR-1AC092535.3
hsa-miR-1RP11-680F20.6
hsa-miR-3LINCMD1
hsa-miR-1DYX1C1-CCPG1
hsa-miR-3RP11-153F5.7
hsa-miR-7LINC01022
hsa-miR-1RP11-22M7.2
hsa-miR-2TTLL10-AS1
hsa-miR-4LINC01128
hsa-miR-1TMEM191A
hsa-miR-1AC011284.3
hsa-miR-2AC015849.16
hsa-miR-1MUC2
hsa-miR-1RP11-867G23.4
hsa-miR-1ADGRA1-AS1
hsa-miR-3AC011284.3
hsa-miR-2TMEM9B-AS1
hsa-miR-1RP11-154D6.1
hsa-miR-7RP11-142C4.6
hsa-miR-2LINC01123
hsa-miR-1CTA-280A3.2
hsa-miR-7LINC01002
hsa-miR-7ST20-AS1
hsa-miR-1RP11-627G23.1
hsa-miR-1CTA-315H11.2
hsa-miR-1RP5-1029F21.2
hsa-miR-1RP11-153F5.7
hsa-miR-1LINC00173
hsa-miR-6RP13-895J2.3
hsa-miR-1RP11-159D12.10
hsa-miR-1RP11-22A3.2
hsa-miR-5LINC00240
hsa-miR-1LINC00265
hsa-miR-1SFTPD-AS1
hsa-miR-1RP11-573D15.2

hsa-miR-1RP11-469N6.1
hsa-miR-1AC015849.13
hsa-miR-1TMEM191A
hsa-miR-1LINC00969
hsa-miR-5CTD-2553C6.1
hsa-miR-5MZF1-AS1
hsa-miR-1AP001062.7
hsa-miR-1RP11-50B3.4
hsa-miR-1RP11-186N15.3
hsa-miR-6RP13-582L3.4
hsa-miR-1H19
hsa-miR-1RP5-1142A6.2
hsa-miR-1RP11-1129I3.1
hsa-miR-1RP11-384K6.6
hsa-miR-5AC005614.3
hsa-miR-5ZNF883
hsa-miR-7CTD-2311B13.1
hsa-miR-1CTA-390C10.9
hsa-miR-5CTC-435M10.10
hsa-miR-1RP11-38M8.1
hsa-miR-7CTC-338M12.9
hsa-miR-1CTD-2245F17.9
hsa-miR-1CTD-3193O13.11
hsa-miR-1RP11-638I8.1
hsa-miR-7AC006019.3
hsa-miR-2RP11-211G23.2
hsa-miR-4ZNF883
hsa-miR-3TTLL10-AS1
hsa-miR-6SNHG14
hsa-miR-7AP001631.9
hsa-miR-7AC005324.6
hsa-miR-1CTD-3193O13.1
hsa-miR-1LINC00689
hsa-miR-1RP13-580B18.4
hsa-miR-6CH17-360D5.1
hsa-miR-7TTN-AS1
hsa-miR-4LINC01067
hsa-miR-3RP11-94C24.13
hsa-miR-3CTD-2619J13.19
hsa-miR-7RP11-394A14.2
hsa-miR-1RP1-29C18.10
hsa-miR-5RP11-762H8.4
hsa-miR-3GS1-279B7.1
hsa-miR-1RP11-483P21.6
hsa-miR-1RP1-288H2.2
hsa-miR-2CTD-2281E23.1
hsa-miR-1CTD-2311B13.1
hsa-miR-8AC079799.2
hsa-miR-1RP11-431K24.1
hsa-miR-1MAFG-AS1
hsa-miR-2CTC-242N15.1

hsa-miR-6LINC00689
hsa-miR-5RP5-1154L15.1
hsa-miR-7ATP2A1-AS1
hsa-miR-1AC124997.1
hsa-miR-1CTD-2008P7.3
hsa-miR-1LINC01168
hsa-miR-5AC137934.1
hsa-miR-6AC114808.3
hsa-miR-5AC006548.28
hsa-miR-3RP11-244B22.11
hsa-miR-3LINCR-0001
hsa-miR-5AC093639.1
hsa-miR-1AC000095.11
hsa-miR-7CTD-3099C6.5
hsa-miR-2NR2F1-AS1
hsa-miR-1RP11-148K1.12
hsa-miR-1AC139099.4
hsa-miR-3LINC00869
hsa-miR-2AC011284.3
hsa-miR-1CTD-2410N18.4
hsa-miR-2CTC-321K16.1
hsa-miR-5SATB1-AS1
hsa-miR-1RP11-430G17.3
hsa-miR-1UCKL1-AS1
hsa-miR-1RP11-1348G14.8
hsa-miR-8RP1-253P7.1
hsa-miR-1AJ003147.8
hsa-miR-5LINC01539
hsa-miR-1COL18A1-AS1
hsa-miR-2LINC01165
hsa-miR-1CTD-2523D13.1
hsa-miR-3LVCAT1
hsa-miR-1CTD-3046C4.1
hsa-miR-1RP11-13K12.1
hsa-miR-1RP11-227H15.4
hsa-miR-1RP3-402G11.28
hsa-miR-1PAX8-AS1
hsa-miR-9RP5-1077H22.2
hsa-miR-7CTD-2008P7.1
hsa-miR-1SSTR5-AS1
hsa-miR-1OR2A1-AS1
hsa-miR-5RP11-598F7.3
hsa-miR-1LINC00662
hsa-miR-5CTD-2561J22.5
hsa-miR-4CTB-60B18.10
hsa-miR-5CTD-2521M24.5
hsa-miR-3RP11-480I12.10
hsa-miR-1CTB-181F24.1
hsa-miR-6RASSF8-AS1
hsa-miR-1LL22NC03-86G7.1
hsa-miR-2LINC00662

hsa-miR-1RP11-630C16.2
hsa-miR-1SNHG14
hsa-miR-7ABHD11-AS1
hsa-miR-1RP11-486P11.1
hsa-miR-5RP11-700J17.1
hsa-miR-4RP4-539M6.22
hsa-miR-5LA16c-60D12.2
hsa-miR-2AC005481.5
hsa-miR-1ZNF833P
hsa-miR-3RP11-374A4.1
hsa-miR-2AC078942.1
hsa-miR-2AC078942.1
hsa-miR-1RP11-458F8.4
hsa-miR-5EGFLAM-AS3
hsa-miR-1MIRLET7BHG
hsa-miR-1AATBC
hsa-miR-4LINC00635
hsa-miR-1CTD-2369P2.8
hsa-miR-7RP11-570L14.2
hsa-miR-2LINC01304
hsa-miR-1CTC-457E21.1
hsa-miR-1SNHG14
hsa-miR-5RP11-638L3.1
hsa-miR-9LA16c-306A4.2
hsa-miR-7RP11-830F9.6
hsa-miR-9RP11-458F8.4
hsa-miR-7RP11-347H15.4
hsa-miR-1RP11-54O7.17
hsa-miR-9RP4-539M6.22
hsa-miR-9CTD-2619J13.14
hsa-miR-1RP11-34P13.7
hsa-miR-9AP001476.4
hsa-miR-9LINC00265

Supplementary file 6: The results of GSVA analyses

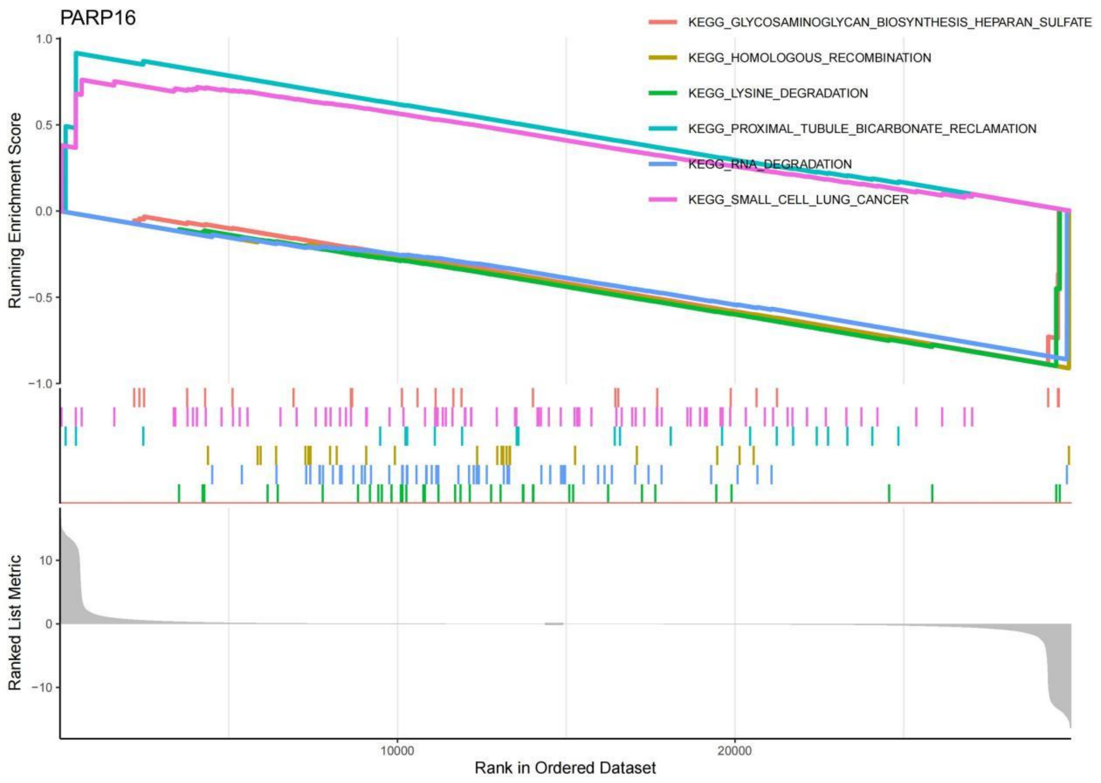

Supplementary file 7a

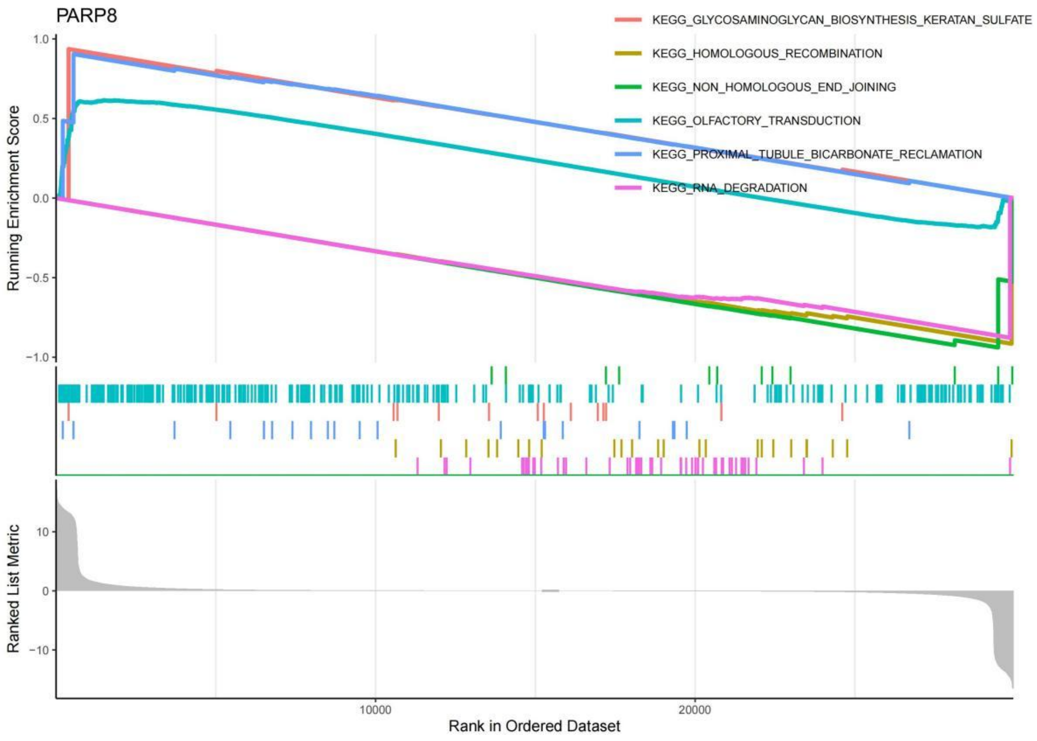

Supplementary file 7b

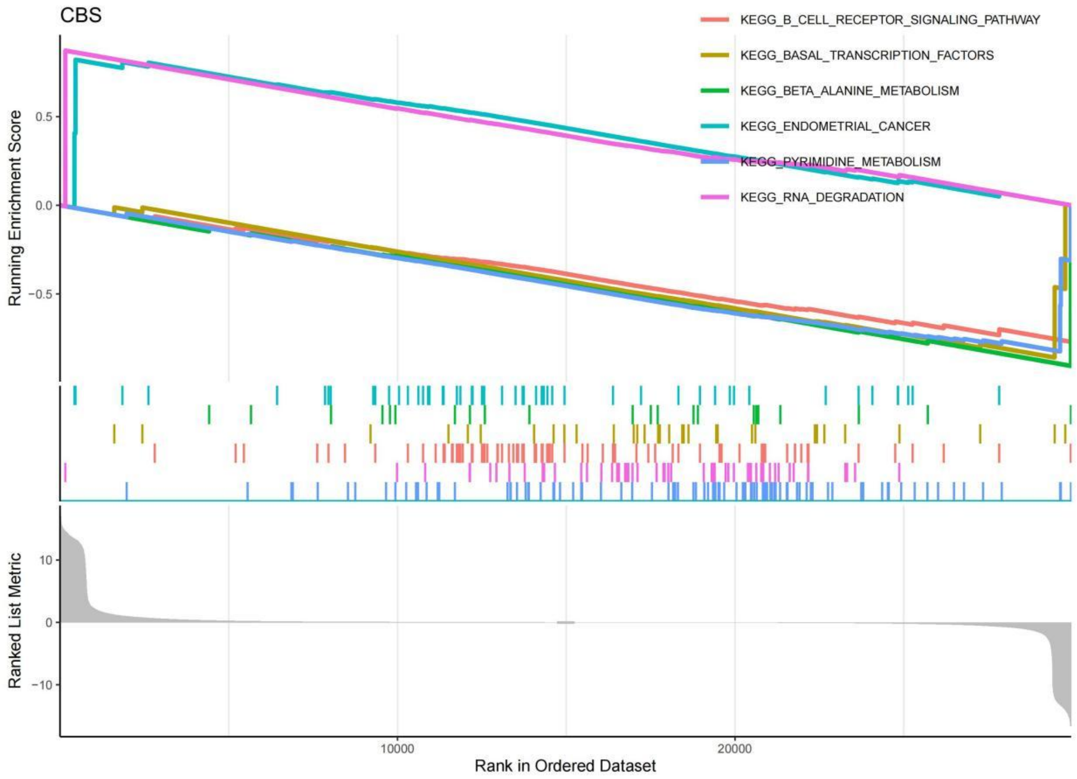

Supplementary file 7c

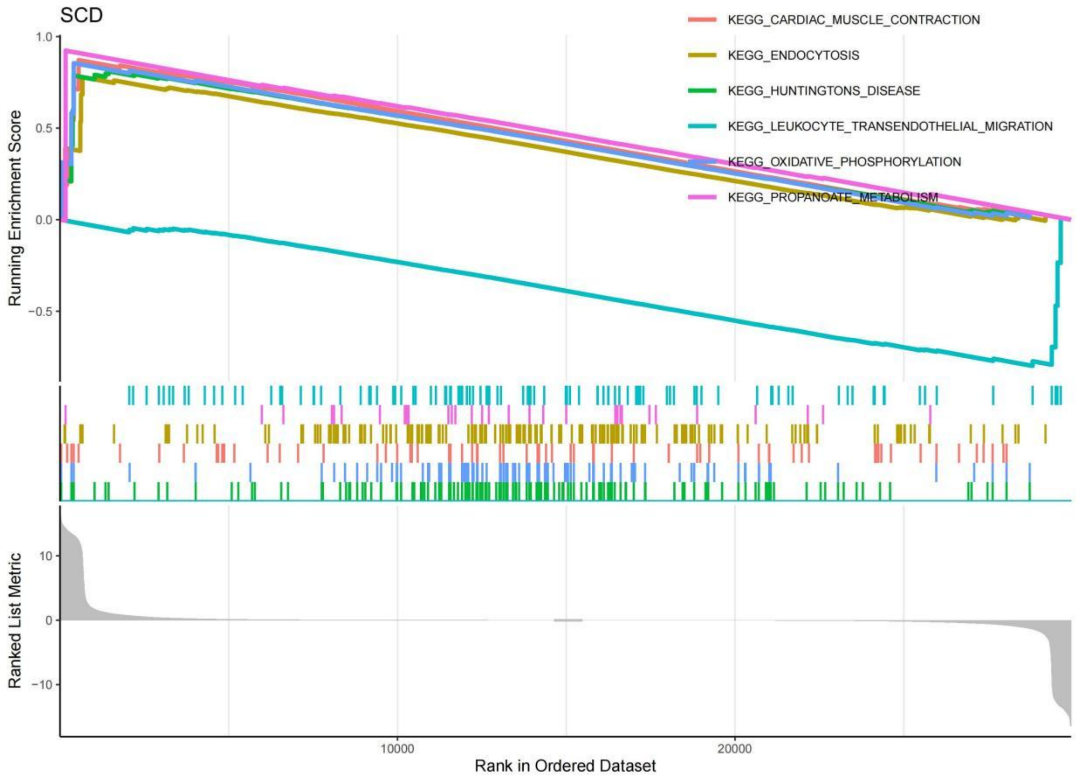

Supplementary file 7d

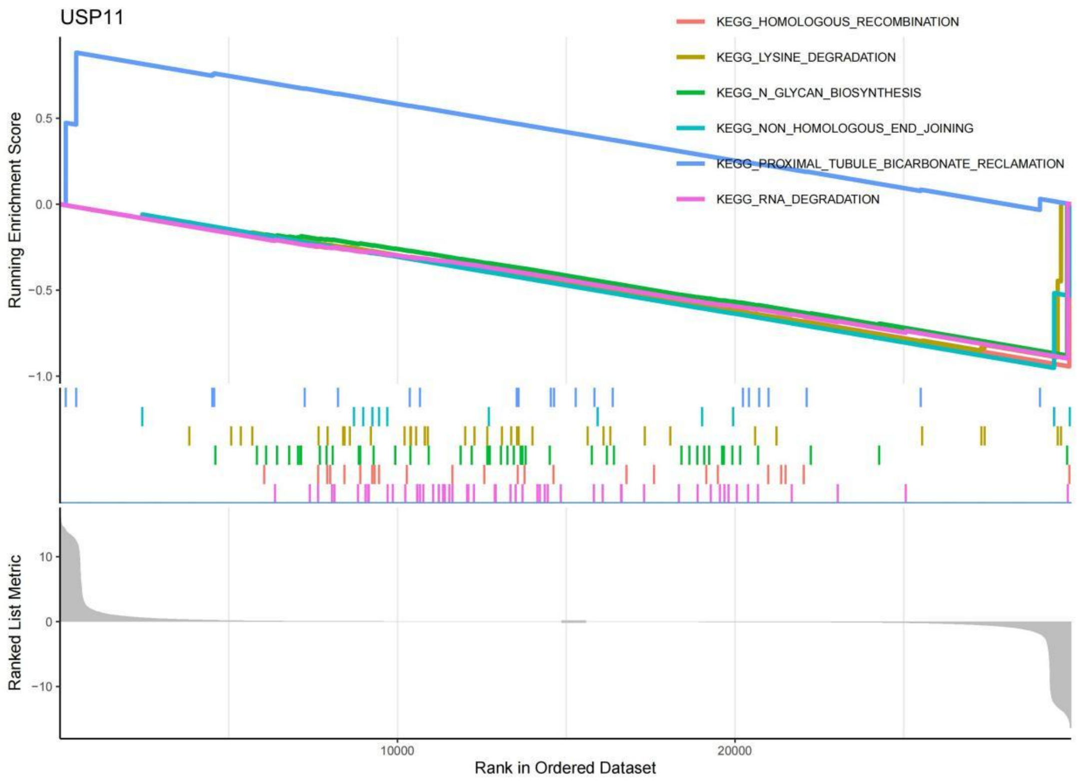

Supplementary file 7e

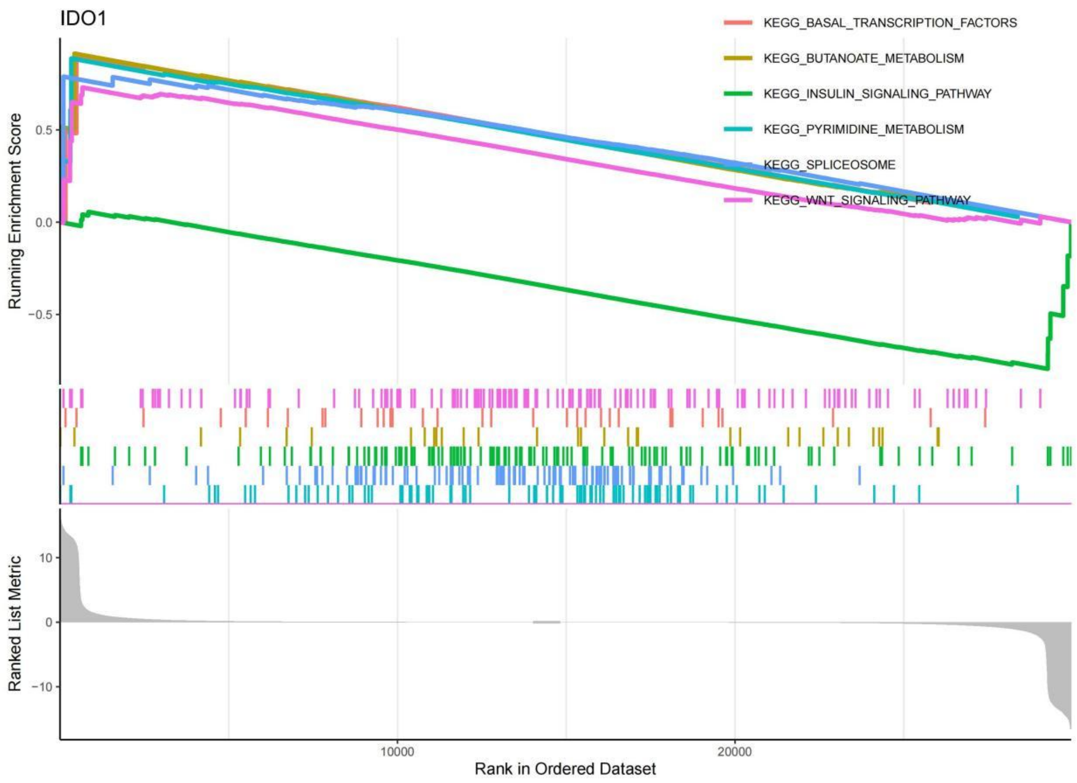

Supplementary file 7f

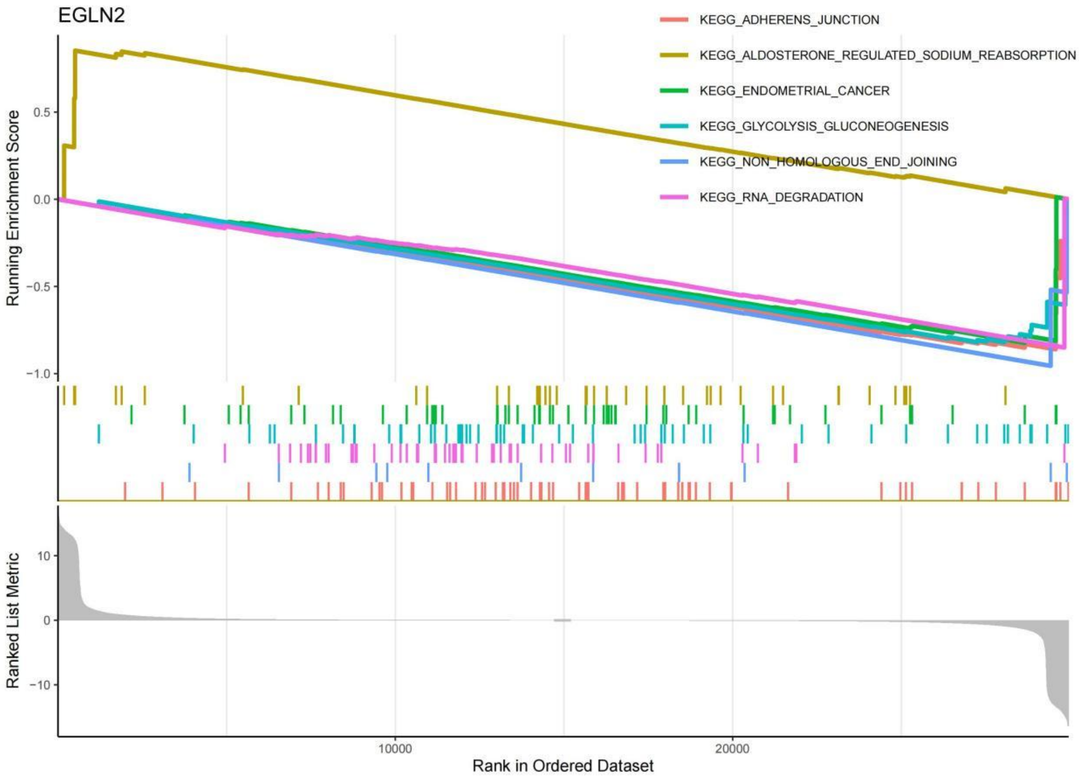

Supplementary file 7g

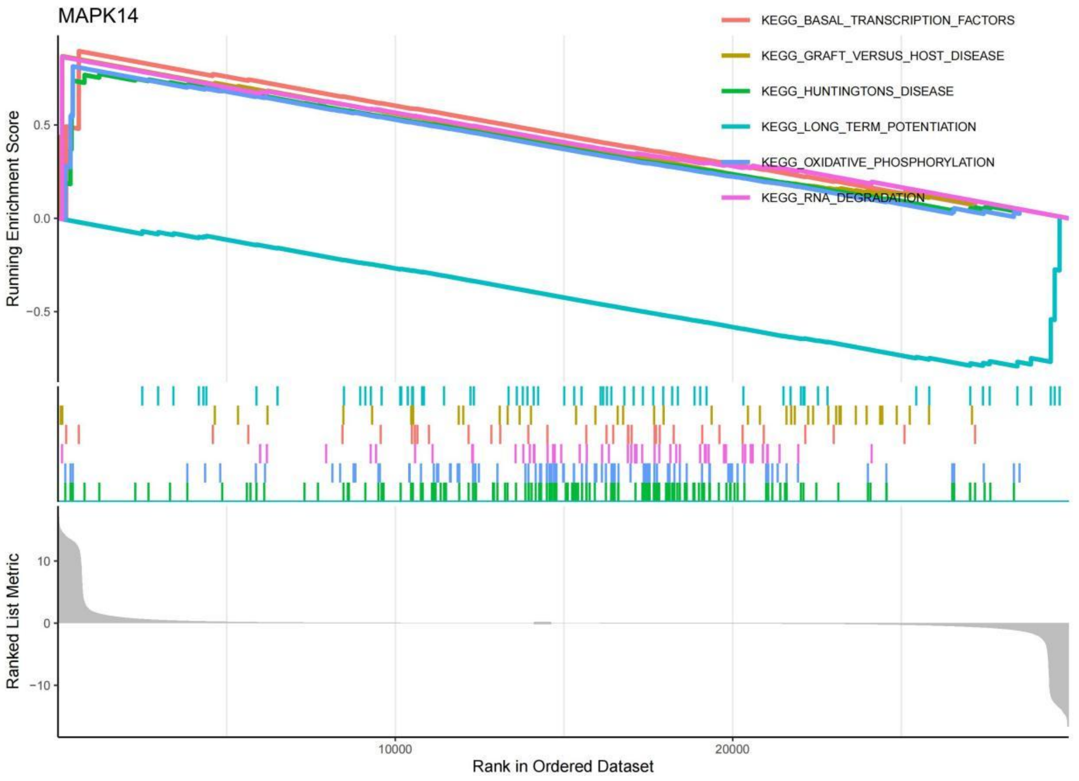

Supplementary file 7h

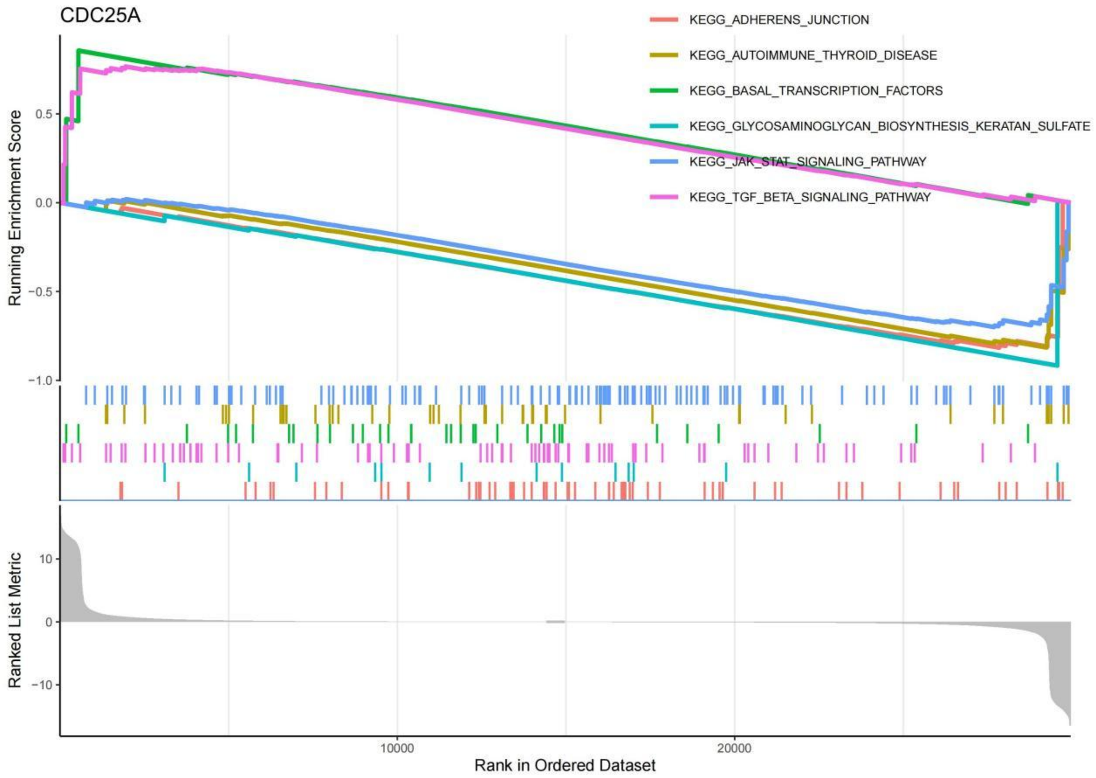

Supplementary file 7 Single-gene GSEA-KEGG pathway analysis in PARP16 (a), PARP8 (b), CBS (c), SCD (d), USP11 (e), IDO1(f), EGLN2 (g), MAPK14 (h) and CDC25A(i).

Supplementary file 7: The details of ceRNA network

Node1	Node2	Interaction
SCD	hsa-miR-1	mRNA
MAPK14	hsa-miR-5	mRNA
PARP8	hsa-miR-3	mRNA
CDC25A	hsa-miR-4	mRNA
CDC25A	hsa-miR-4	mRNA
SCD	hsa-miR-3	mRNA
CBS	hsa-miR-1	mRNA
CDC25A	hsa-miR-3	mRNA
SCD	hsa-miR-9	mRNA
MAPK14	hsa-miR-3	mRNA
PARP16	hsa-miR-5	mRNA
SCD	hsa-miR-6	mRNA
PARP8	hsa-miR-3	mRNA
EGLN2	hsa-miR-1	mRNA
SCD	hsa-miR-6	mRNA
PARP16	hsa-miR-5	mRNA
EGLN2	hsa-let-7	mRNA
MAPK14	hsa-miR-1	mRNA
SCD	hsa-miR-1	mRNA
SCD	hsa-miR-6	mRNA
SCD	hsa-miR-1	mRNA
PARP16	hsa-miR-5	mRNA
EGLN2	hsa-let-7	mRNA
MAPK14	hsa-miR-2	mRNA
SCD	hsa-miR-2	mRNA
CDC25A	hsa-miR-1	mRNA
CDC25A	hsa-miR-6	mRNA
CDC25A	hsa-miR-1	mRNA
CDC25A	hsa-miR-4	mRNA
CDC25A	hsa-miR-7	mRNA
SCD	hsa-miR-5	mRNA
PARP8	hsa-miR-5	mRNA
MAPK14	hsa-miR-3	mRNA
MAPK14	hsa-miR-2	mRNA
MAPK14	hsa-miR-1	mRNA
MAPK14	hsa-miR-1	mRNA
PARP8	hsa-miR-4	mRNA
SCD	hsa-miR-5	mRNA
PARP8	hsa-miR-4	mRNA
EGLN2	hsa-miR-1	mRNA
EGLN2	hsa-miR-5	mRNA
SCD	hsa-miR-1	mRNA
CDC25A	hsa-miR-4	mRNA
CBS	hsa-miR-4	mRNA
CBS	hsa-miR-4	mRNA
SCD	hsa-miR-1	mRNA
MAPK14	hsa-miR-4	mRNA
SCD	hsa-miR-2	mRNA
PARP8	hsa-miR-3	mRNA

EGLN2	hsa-miR-3mRNA
CDC25A	hsa-let-7mRNA
PARP16	hsa-miR-1mRNA
SCD	hsa-miR-4mRNA
CDC25A	hsa-miR-4mRNA
CDC25A	hsa-miR-1mRNA
SCD	hsa-let-7mRNA
CDC25A	hsa-miR-4mRNA
CDC25A	hsa-miR-4mRNA
PARP8	hsa-miR-3mRNA
MAPK14	hsa-miR-4mRNA
SCD	hsa-miR-2mRNA
MAPK14	hsa-miR-7mRNA
PARP16	hsa-miR-1mRNA
CDC25A	hsa-miR-1mRNA
CDC25A	hsa-miR-1mRNA
PARP8	hsa-miR-3mRNA
CDC25A	hsa-let-7mRNA
MAPK14	hsa-miR-4mRNA
PARP16	hsa-miR-6mRNA
CDC25A	hsa-miR-1mRNA
MAPK14	hsa-miR-1mRNA
SCD	hsa-miR-1mRNA
CBS	hsa-miR-1mRNA
MAPK14	hsa-miR-4mRNA
SCD	hsa-miR-3mRNA
PARP16	hsa-miR-3mRNA
PARP8	hsa-miR-3mRNA
CBS	hsa-miR-1mRNA
PARP16	hsa-miR-5mRNA
MAPK14	hsa-miR-7mRNA
EGLN2	hsa-let-7mRNA
SCD	hsa-miR-1mRNA
SCD	hsa-miR-5mRNA
PARP16	hsa-miR-3mRNA
PARP16	hsa-miR-3mRNA
MAPK14	hsa-miR-3mRNA
SCD	hsa-miR-4mRNA
CDC25A	hsa-miR-3mRNA
MAPK14	hsa-miR-5mRNA
PARP8	hsa-miR-3mRNA
CDC25A	hsa-miR-4mRNA
SCD	hsa-miR-1mRNA
CBS	hsa-miR-6mRNA
SCD	hsa-miR-4mRNA
PARP16	hsa-miR-2mRNA
PARP8	hsa-miR-5mRNA
SCD	hsa-miR-1mRNA
PARP16	hsa-miR-5mRNA
CDC25A	hsa-miR-1mRNA
PARP8	hsa-miR-5mRNA

USP11	hsa-miR-1mRNA
CDC25A	hsa-let-7mRNA
SCD	hsa-let-7mRNA
PARP16	hsa-miR-5mRNA
PARP16	hsa-miR-2mRNA
SCD	hsa-miR-3mRNA
PARP16	hsa-miR-6mRNA
CDC25A	hsa-miR-6mRNA
EGLN2	hsa-let-7mRNA
PARP8	hsa-miR-6mRNA
PARP16	hsa-miR-3mRNA
EGLN2	hsa-miR-4mRNA
EGLN2	hsa-let-7mRNA
CDC25A	hsa-miR-1mRNA
MAPK14	hsa-miR-1mRNA
SCD	hsa-miR-4mRNA
PARP16	hsa-miR-7mRNA
PARP16	hsa-miR-5mRNA
CDC25A	hsa-let-7mRNA
PARP8	hsa-miR-4mRNA
PARP16	hsa-miR-4mRNA
CDC25A	hsa-miR-2mRNA
PARP8	hsa-miR-1mRNA
PARP16	hsa-miR-3mRNA
PARP8	hsa-miR-9mRNA
PARP8	hsa-miR-5mRNA
PARP16	hsa-miR-4mRNA
PARP8	hsa-miR-3mRNA
CDC25A	hsa-miR-1mRNA
SCD	hsa-miR-3mRNA
SCD	hsa-let-7mRNA
CDC25A	hsa-miR-1mRNA
SCD	hsa-miR-1mRNA
EGLN2	hsa-miR-9mRNA
SCD	hsa-miR-6mRNA
SCD	hsa-miR-4mRNA
SCD	hsa-miR-3mRNA
SCD	hsa-miR-5mRNA
PARP16	hsa-miR-5mRNA
CDC25A	hsa-miR-3mRNA
SCD	hsa-miR-1mRNA
SCD	hsa-miR-1mRNA
SCD	hsa-let-7mRNA
EGLN2	hsa-miR-9mRNA
MAPK14	hsa-miR-1mRNA
PARP16	hsa-miR-1mRNA
CDC25A	hsa-miR-1mRNA
PARP8	hsa-miR-2mRNA
MAPK14	hsa-miR-4mRNA
MAPK14	hsa-miR-1mRNA
MAPK14	hsa-miR-2mRNA

CDC25A	hsa-miR-5	mRNA
SCD	hsa-miR-9	mRNA
PARP16	hsa-miR-5	mRNA
USP11	hsa-miR-5	mRNA
SCD	hsa-miR-1	mRNA
SCD	hsa-miR-1	mRNA
SCD	hsa-miR-9	mRNA
EGLN2	hsa-miR-3	mRNA
PARP8	hsa-miR-5	mRNA
MAPK14	hsa-miR-3	mRNA
MAPK14	hsa-miR-2	mRNA
EGLN2	hsa-miR-2	mRNA
EGLN2	hsa-miR-2	mRNA
SCD	hsa-miR-3	mRNA
MAPK14	hsa-miR-7	mRNA
IDO1	hsa-miR-5	mRNA
MAPK14	hsa-miR-3	mRNA
CDC25A	hsa-miR-3	mRNA
SCD	hsa-miR-3	mRNA
SCD	hsa-miR-1	mRNA
SCD	hsa-miR-8	mRNA
CBS	hsa-miR-1	mRNA
CBS	hsa-miR-2	mRNA
CDC25A	hsa-let-7	mRNA
MAPK14	hsa-miR-5	mRNA
CDC25A	hsa-miR-4	mRNA
PARP8	hsa-miR-4	mRNA
SCD	hsa-miR-1	mRNA
IDO1	hsa-miR-4	mRNA
PARP16	hsa-miR-1	mRNA
CDC25A	hsa-miR-1	mRNA
EGLN2	hsa-miR-4	mRNA
IDO1	hsa-miR-1	mRNA
PARP16	hsa-miR-3	mRNA
SCD	hsa-miR-1	mRNA
CDC25A	hsa-miR-3	mRNA
SCD	hsa-miR-1	mRNA
MAPK14	hsa-miR-5	mRNA
MAPK14	hsa-miR-1	mRNA
MAPK14	hsa-miR-5	mRNA
SCD	hsa-miR-5	mRNA
CBS	hsa-miR-4	mRNA
PARP8	hsa-miR-2	mRNA
CDC25A	hsa-miR-1	mRNA
CDC25A	hsa-miR-6	mRNA
SCD	hsa-miR-6	mRNA
PARP16	hsa-miR-2	mRNA
SCD	hsa-miR-7	mRNA
SCD	hsa-miR-5	mRNA
MAPK14	hsa-miR-5	mRNA
MAPK14	hsa-miR-3	mRNA

SCD	hsa-miR-9	mRNA
MAPK14	hsa-miR-4	mRNA
MAPK14	hsa-miR-2	mRNA
CDC25A	hsa-miR-2	mRNA
SCD	hsa-miR-1	mRNA
SCD	hsa-miR-4	mRNA
PARP16	hsa-miR-3	mRNA
EGLN2	hsa-let-7	mRNA
SCD	hsa-miR-1	mRNA
CDC25A	hsa-miR-2	mRNA
PARP8	hsa-miR-3	mRNA
CDC25A	hsa-miR-1	mRNA
PARP16	hsa-miR-4	mRNA
MAPK14	hsa-miR-4	mRNA
MAPK14	hsa-miR-5	mRNA
PARP8	hsa-miR-5	mRNA
SCD	hsa-let-7	mRNA
PARP8	hsa-miR-5	mRNA
CDC25A	hsa-miR-3	mRNA
MAPK14	hsa-miR-6	mRNA
SCD	hsa-miR-9	mRNA
SCD	hsa-miR-9	mRNA
PARP8	hsa-miR-5	mRNA
SCD	hsa-miR-1	mRNA
SCD	hsa-let-7	mRNA
CDC25A	hsa-miR-9	mRNA
SCD	hsa-miR-5	mRNA
MAPK14	hsa-miR-1	mRNA
PARP8	hsa-miR-3	mRNA
SCD	hsa-miR-3	mRNA
CDC25A	hsa-miR-1	mRNA
SCD	hsa-miR-1	mRNA
CBS	hsa-miR-4	mRNA
CDC25A	hsa-let-7	mRNA
EGLN2	hsa-miR-2	mRNA
EGLN2	hsa-miR-1	mRNA
EGLN2	hsa-let-7	mRNA
MAPK14	hsa-miR-3	mRNA
EGLN2	hsa-miR-6	mRNA
IDO1	hsa-miR-5	mRNA
PARP16	hsa-miR-5	mRNA
SCD	hsa-miR-1	mRNA
CDC25A	hsa-miR-9	mRNA
EGLN2	hsa-miR-4	mRNA
CDC25A	hsa-miR-3	mRNA
PARP8	hsa-miR-5	mRNA
CDC25A	hsa-miR-5	mRNA
IDO1	hsa-miR-3	mRNA
CDC25A	hsa-miR-1	mRNA
IDO1	hsa-miR-7	mRNA
MAPK14	hsa-miR-5	mRNA

EGLN2	hsa-miR-5	mRNA
PARP16	hsa-miR-5	mRNA
MAPK14	hsa-miR-6	mRNA
EGLN2	hsa-miR-1	mRNA
EGLN2	hsa-miR-4	mRNA
EGLN2	hsa-miR-7	mRNA
CDC25A	hsa-let-7	mRNA
PARP8	hsa-miR-5	mRNA
PARP8	hsa-miR-3	mRNA
PARP8	hsa-miR-1	mRNA
SCD	hsa-miR-6	mRNA
MAPK14	hsa-miR-1	mRNA
SCD	hsa-miR-1	mRNA
PARP16	hsa-miR-8	mRNA
SCD	hsa-miR-4	mRNA
PARP8	hsa-miR-3	mRNA
PARP16	hsa-miR-4	mRNA
CDC25A	hsa-miR-5	mRNA
SCD	hsa-let-7	mRNA
LINC01043	hsa-miR-1	lncRNA
GAS6-AS1	hsa-miR-7	lncRNA
HP09025	hsa-miR-1	lncRNA
LA16c-306	hsa-miR-2	lncRNA
RP11-830F	hsa-miR-1	lncRNA
RP11-10J2	hsa-miR-2	lncRNA
RP5-894D1	hsa-miR-6	lncRNA
RP5-894D1	hsa-miR-6	lncRNA
RP1-34P24	hsa-miR-6	lncRNA
C10orf91	hsa-miR-3	lncRNA
C10orf91	hsa-miR-1	lncRNA
MUC19	hsa-miR-1	lncRNA
AC079779.	hsa-miR-4	lncRNA
LINC01043	hsa-miR-1	lncRNA
RP11-102K	hsa-miR-2	lncRNA
LINC01070	hsa-miR-5	lncRNA
RP11-982M	hsa-miR-5	lncRNA
C10orf91	hsa-miR-4	lncRNA
RP11-627G	hsa-miR-6	lncRNA
CTC-265F1	hsa-miR-1	lncRNA
LINC01070	hsa-miR-7	lncRNA
RP11-138E	hsa-miR-7	lncRNA
RP11-982M	hsa-miR-1	lncRNA
AC092657.	hsa-miR-1	lncRNA
RP11-99L1	hsa-miR-1	lncRNA
CTB-186H2	hsa-miR-1	lncRNA
TMEM191C	hsa-miR-1	lncRNA
RP11-326C	hsa-miR-2	lncRNA
LINC01106	hsa-miR-2	lncRNA
RP11-830F	hsa-miR-1	lncRNA
RP3-388N1	hsa-miR-1	lncRNA
RP4-737E2	hsa-miR-4	lncRNA

RP11-138E	hsa-miR-4	lncRNA
RP11-333E	hsa-miR-1	lncRNA
RP4-539M6	hsa-miR-2	lncRNA
AC011284.	hsa-miR-1	lncRNA
AATBC	hsa-miR-1	lncRNA
RP11-627G	hsa-miR-7	lncRNA
RP13-507F	hsa-miR-7	lncRNA
LA16c-306	hsa-miR-3	lncRNA
LINC01002	hsa-miR-1	lncRNA
LINC01002	hsa-miR-1	lncRNA
AP001476.	hsa-miR-3	lncRNA
MIR325HG	hsa-miR-1	lncRNA
C10orf91	hsa-miR-1	lncRNA
COL4A2-AS	hsa-miR-1	lncRNA
LINC00689	hsa-miR-1	lncRNA
RP11-618K	hsa-miR-1	lncRNA
GAS8-AS1	hsa-miR-1	lncRNA
RP11-717I	hsa-miR-5	lncRNA
RP11-5407	hsa-miR-1	lncRNA
MUC19	hsa-miR-7	lncRNA
LINC00689	hsa-miR-8	lncRNA
RP11-326C	hsa-miR-2	lncRNA
HPVC1	hsa-miR-5	lncRNA
CTA-941F9	hsa-miR-1	lncRNA
VIPR1-AS1	hsa-miR-4	lncRNA
RP11-394A	hsa-miR-1	lncRNA
CTD-2008F	hsa-miR-7	lncRNA
MUC2	hsa-miR-3	lncRNA
LINC00265	hsa-miR-1	lncRNA
RP11-333E	hsa-miR-1	lncRNA
RP13-580E	hsa-miR-7	lncRNA
LINC00685	hsa-miR-1	lncRNA
LINC01165	hsa-miR-1	lncRNA
RP11-32B5	hsa-miR-1	lncRNA
AIRN	hsa-miR-1	lncRNA
RP3-470B2	hsa-miR-1	lncRNA
AC084219.	hsa-miR-1	lncRNA
AC078942.	hsa-miR-7	lncRNA
RP11-210M	hsa-miR-4	lncRNA
FAM230B	hsa-miR-1	lncRNA
CTB-51J22	hsa-miR-6	lncRNA
RP4-539M6	hsa-miR-3	lncRNA
RP11-94C2	hsa-let-7	lncRNA
RP11-94C2	hsa-miR-4	lncRNA
AP001476.	hsa-miR-1	lncRNA
RP4-539M6	hsa-miR-1	lncRNA
LL22NC03-	hsa-miR-1	lncRNA
CTD-2619J	hsa-miR-1	lncRNA
RP11-384K	hsa-miR-1	lncRNA
FAM182A	hsa-miR-4	lncRNA
HCG22	hsa-miR-1	lncRNA

CTD-2561E	hsa-miR-6	lncRNA
LINC01165	hsa-miR-4	lncRNA
RP1-182D1	hsa-miR-5	lncRNA
FLJ35934	hsa-miR-1	lncRNA
HPVC1	hsa-miR-1	lncRNA
LINC01165	hsa-miR-5	lncRNA
LINC00265	hsa-miR-1	lncRNA
LINC01123	hsa-miR-1	lncRNA
RP11-311F	hsa-miR-1	lncRNA
CTD-2532K	hsa-miR-5	lncRNA
RP11-426C	hsa-miR-1	lncRNA
RP11-1217	hsa-miR-7	lncRNA
CH507-216	hsa-miR-4	lncRNA
NNT-AS1	hsa-miR-1	lncRNA
GS1-251I9	hsa-miR-1	lncRNA
CTD-3193C	hsa-miR-7	lncRNA
LINC00689	hsa-miR-1	lncRNA
LINC00940	hsa-miR-8	lncRNA
CTD-3099C	hsa-miR-1	lncRNA
LINC00613	hsa-miR-1	lncRNA
LINC01043	hsa-miR-2	lncRNA
RP5-1029F	hsa-miR-6	lncRNA
CTD-2517M	hsa-miR-1	lncRNA
RP11-269G	hsa-miR-1	lncRNA
RP11-762H	hsa-miR-6	lncRNA
RP11-449D	hsa-miR-2	lncRNA
RP11-126C	hsa-miR-1	lncRNA
RP11-126C	hsa-miR-1	lncRNA
RP11-333E	hsa-miR-2	lncRNA
CTD-2008F	hsa-miR-1	lncRNA
RP11-85G1	hsa-miR-7	lncRNA
AC018816.	hsa-miR-5	lncRNA
AC005330.	hsa-miR-5	lncRNA
AC092535.	hsa-miR-1	lncRNA
RP11-680F	hsa-miR-1	lncRNA
LINCMD1	hsa-miR-3	lncRNA
DYX1C1-CC	hsa-miR-1	lncRNA
RP11-153F	hsa-miR-3	lncRNA
LINC01022	hsa-miR-7	lncRNA
RP11-22M7	hsa-miR-1	lncRNA
TTLL10-AS	hsa-miR-2	lncRNA
LINC01128	hsa-miR-4	lncRNA
TMEM191A	hsa-miR-1	lncRNA
AC011284.	hsa-miR-1	lncRNA
AC015849.	hsa-miR-2	lncRNA
MUC2	hsa-miR-1	lncRNA
RP11-867G	hsa-miR-1	lncRNA
ADGRA1-AS	hsa-miR-1	lncRNA
AC011284.	hsa-miR-3	lncRNA
TMEM9B-AS	hsa-miR-2	lncRNA
RP11-154D	hsa-miR-1	lncRNA

RP11-142C	hsa-miR-7	lncRNA
LINC01123	hsa-miR-2	lncRNA
CTA-280A3	hsa-miR-1	lncRNA
LINC01002	hsa-miR-7	lncRNA
ST20-AS1	hsa-miR-7	lncRNA
RP11-627G	hsa-miR-1	lncRNA
CTA-315H1	hsa-miR-1	lncRNA
RP5-1029F	hsa-miR-1	lncRNA
RP11-153F	hsa-miR-1	lncRNA
LINC00173	hsa-miR-1	lncRNA
RP13-895J	hsa-miR-6	lncRNA
RP11-159D	hsa-miR-1	lncRNA
RP11-22A3	hsa-miR-1	lncRNA
LINC00240	hsa-miR-5	lncRNA
LINC00265	hsa-miR-1	lncRNA
SFTPD-AS1	hsa-miR-1	lncRNA
RP11-573D	hsa-miR-1	lncRNA
RP11-469N	hsa-miR-1	lncRNA
AC015849.	hsa-miR-1	lncRNA
TMEM191A	hsa-miR-1	lncRNA
LINC00969	hsa-miR-1	lncRNA
CTD-2553C	hsa-miR-5	lncRNA
MZF1-AS1	hsa-miR-5	lncRNA
AP001062.	hsa-miR-1	lncRNA
RP11-50B3	hsa-miR-1	lncRNA
RP11-186N	hsa-miR-1	lncRNA
RP13-582L	hsa-miR-6	lncRNA
H19	hsa-miR-1	lncRNA
RP5-1142A	hsa-miR-1	lncRNA
RP11-1129	hsa-miR-1	lncRNA
RP11-384K	hsa-miR-1	lncRNA
AC005614.	hsa-miR-5	lncRNA
ZNF883	hsa-miR-5	lncRNA
CTD-2311E	hsa-miR-7	lncRNA
CTA-390C1	hsa-miR-1	lncRNA
CTC-435M1	hsa-miR-5	lncRNA
RP11-38M8	hsa-miR-1	lncRNA
CTC-338M1	hsa-miR-7	lncRNA
CTD-2245F	hsa-miR-1	lncRNA
CTD-3193C	hsa-miR-1	lncRNA
RP11-638I	hsa-miR-1	lncRNA
AC006019.	hsa-miR-7	lncRNA
RP11-211G	hsa-miR-2	lncRNA
ZNF883	hsa-miR-4	lncRNA
TTLL10-AS	hsa-miR-3	lncRNA
SNHG14	hsa-miR-6	lncRNA
AP001631.	hsa-miR-7	lncRNA
AC005324.	hsa-miR-7	lncRNA
CTD-3193C	hsa-miR-1	lncRNA
LINC00689	hsa-miR-1	lncRNA
RP13-580E	hsa-miR-1	lncRNA

CH17-360D	hsa-miR-6	lncRNA
TTN-AS1	hsa-miR-7	lncRNA
LINC01067	hsa-miR-4	lncRNA
RP11-94C2	hsa-miR-3	lncRNA
CTD-2619J	hsa-miR-3	lncRNA
RP11-394A	hsa-miR-7	lncRNA
RP1-29C18	hsa-miR-1	lncRNA
RP11-762H	hsa-miR-5	lncRNA
GS1-279B7	hsa-miR-3	lncRNA
RP11-483F	hsa-miR-1	lncRNA
RP1-288H2	hsa-miR-1	lncRNA
CTD-2281E	hsa-miR-2	lncRNA
CTD-2311E	hsa-miR-1	lncRNA
AC079799.	hsa-miR-8	lncRNA
RP11-431K	hsa-miR-1	lncRNA
MAFG-AS1	hsa-miR-1	lncRNA
CTC-242N1	hsa-miR-2	lncRNA
LINC00689	hsa-miR-6	lncRNA
RP5-1154L	hsa-miR-5	lncRNA
ATP2A1-AS	hsa-miR-7	lncRNA
AC124997.	hsa-miR-1	lncRNA
CTD-2008F	hsa-miR-1	lncRNA
LINC01168	hsa-miR-1	lncRNA
AC137934.	hsa-miR-5	lncRNA
AC114808.	hsa-miR-6	lncRNA
AC006548.	hsa-miR-5	lncRNA
RP11-244E	hsa-miR-3	lncRNA
LINCR-00C	hsa-miR-3	lncRNA
AC093639.	hsa-miR-5	lncRNA
AC000095.	hsa-miR-1	lncRNA
CTD-3099C	hsa-miR-7	lncRNA
NR2F1-AS1	hsa-miR-2	lncRNA
RP11-148K	hsa-miR-1	lncRNA
AC139099.	hsa-miR-1	lncRNA
LINC00869	hsa-miR-3	lncRNA
AC011284.	hsa-miR-2	lncRNA
CTD-2410N	hsa-miR-1	lncRNA
CTC-321K1	hsa-miR-2	lncRNA
SATB1-AS1	hsa-miR-5	lncRNA
RP11-430G	hsa-miR-1	lncRNA
UCKL1-AS1	hsa-miR-1	lncRNA
RP11-1348	hsa-miR-1	lncRNA
RP1-253P7	hsa-miR-8	lncRNA
AJ003147.	hsa-miR-1	lncRNA
LINC01539	hsa-miR-5	lncRNA
COL18A1-A	hsa-miR-1	lncRNA
LINC01165	hsa-miR-2	lncRNA
CTD-2523D	hsa-miR-1	lncRNA
LVCAT1	hsa-miR-3	lncRNA
CTD-3046C	hsa-miR-1	lncRNA
RP11-13K1	hsa-miR-1	lncRNA

RP11-227H	hsa-miR-1	lncRNA
RP3-402G1	hsa-miR-1	lncRNA
PAX8-AS1	hsa-miR-1	lncRNA
RP5-1077H	hsa-miR-9	lncRNA
CTD-2008P	hsa-miR-7	lncRNA
SSTR5-AS1	hsa-miR-1	lncRNA
OR2A1-AS1	hsa-miR-1	lncRNA
RP11-598F	hsa-miR-5	lncRNA
LINC00662	hsa-miR-1	lncRNA
CTD-2561J	hsa-miR-5	lncRNA
CTB-60B18	hsa-miR-4	lncRNA
CTD-2521M	hsa-miR-5	lncRNA
RP11-480I	hsa-miR-3	lncRNA
CTB-181F2	hsa-miR-1	lncRNA
RASSF8-AS	hsa-miR-6	lncRNA
LL22NC03-	hsa-miR-1	lncRNA
LINC00662	hsa-miR-2	lncRNA
RP11-630C	hsa-miR-1	lncRNA
SNHG14	hsa-miR-1	lncRNA
ABHD11-AS	hsa-miR-7	lncRNA
RP11-486F	hsa-miR-1	lncRNA
RP11-700J	hsa-miR-5	lncRNA
RP4-539M6	hsa-miR-4	lncRNA
LA16c-60D	hsa-miR-5	lncRNA
AC005481.	hsa-miR-2	lncRNA
ZNF833P	hsa-miR-1	lncRNA
RP11-374A	hsa-miR-3	lncRNA
AC078942.	hsa-miR-2	lncRNA
AC078942.	hsa-miR-2	lncRNA
RP11-458F	hsa-miR-1	lncRNA
EGFLAM-AS	hsa-miR-5	lncRNA
MIRLET7BH	hsa-miR-1	lncRNA
AATBC	hsa-miR-1	lncRNA
LINC00635	hsa-miR-4	lncRNA
CTD-2369P	hsa-miR-1	lncRNA
RP11-570L	hsa-miR-7	lncRNA
LINC01304	hsa-miR-2	lncRNA
CTC-457E2	hsa-miR-1	lncRNA
SNHG14	hsa-miR-1	lncRNA
RP11-638L	hsa-miR-5	lncRNA
LA16c-306	hsa-miR-9	lncRNA
RP11-830F	hsa-miR-7	lncRNA
RP11-458F	hsa-miR-9	lncRNA
RP11-347H	hsa-miR-7	lncRNA
RP11-5407	hsa-miR-1	lncRNA
RP4-539M6	hsa-miR-9	lncRNA
CTD-2619J	hsa-miR-9	lncRNA
RP11-34P1	hsa-miR-1	lncRNA
AP001476.	hsa-miR-9	lncRNA
LINC00265	hsa-miR-9	lncRNA
